# Genomic characterization of remission in juvenile idiopathic arthritis

**DOI:** 10.1186/ar4280

**Published:** 2013-08-30

**Authors:** Kaiyu Jiang, Mark Barton Frank, Yanmin Chen, Jeanette Osban, James N Jarvis

**Affiliations:** 1 Department of Pediatrics, Pediatric Rheumatology Research, University of Oklahoma Health Sciences Center, Basic Sciences Education Building 235A, Oklahoma City, OK 73104, USA; 2 Microarray Research Facility, Arthritis and Clinical Immunology Program, Oklahoma Medical Research Foundation, 840 NE 13th Street, Oklahoma City, OK 73104, USA; 3 Department of Pediatrics, Rheumatology Research, SUNY Buffalo Clinical and Translational Research Center, 875 Ellicott Street, Buffalo, NY 14203

**Keywords:** juvenile idiopathic arthritis, methotrexate, TNF inhibitor, gene expression, biomarker, microarray

## Abstract

**Introduction:**

The attainment of remission has become an important end point for clinical trials in juvenile idiopathic arthritis (JIA), although we do not yet have a full understanding of what remission is at the cell and molecular level.

**Methods:**

Two independent cohorts of patients with JIA and healthy child controls were studied. RNA was prepared separately from peripheral blood mononuclear cells (PBMC) and granulocytes to identify differentially expressed genes using whole genome microarrays. Expression profiling results for selected genes were confirmed by quantitative, real-time polymerase chain reaction (RT-PCR).

**Results:**

We found that remission in JIA induced by either methotrexate (MTX) or MTX plus a TNF inhibitor (etanercept, Et) (MTX + Et) is characterized by numerous differences in gene expression in peripheral blood mononuclear cells and in granulocytes compared with healthy control children; that is, remission is not a restoration of immunologic normalcy. Network analysis of the differentially expressed genes demonstrated that the steroid hormone receptor superfamily member hepatocyte nuclear factor 4 alpha (HNF4α) is a hub in several of the gene networks that distinguished children with arthritis from controls. Confocal microscopy revealed that HNF4a is present in both T lymphocytes and granulocytes, suggesting a previously unsuspected role for this transcription factor in regulating leukocyte function and therapeutic response in JIA.

**Conclusions:**

These findings provide a framework from which to understand therapeutic response in JIA and, furthermore, may be used to develop strategies to increase the frequency with which remission is achieved in adult forms of rheumatoid arthritis.

## Introduction

The advent of biological therapies for chronic forms of arthritis has been accompanied by the hopes that: (1) therapies can be increasingly tailored to specific pathogenic pathways, decreasing unwanted side effects; and (2) by use of more targeted therapies, patients will experience more sustained periods of disease quiescence and, therefore, functional and subjective well-being. In juvenile idiopathic arthritis (JIA), the most common form of chronic arthritis in children, achieving the second of these objectives appears to be very near [1].

JIA is a term used to denote a heterogeneous group of childhood illnesses characterized by chronic inflammation and hypertrophy of synovial membranes. Distinct phenotypes are recognized based on disease presentation, clinical course, and specific biomarkers, for example, IgM rheumatoid factor [2]. However, even within carefully specified disease subtypes, considerable heterogeneity exists, especially with respect to response to therapy and overall outcome [3]. The biology underlying these differences is poorly understood, and obtaining a molecular understanding of phenotypic and therapeutic response differences is an important step toward developing individualized therapies for this family of diseases and their cognate conditions in adults.

A major advance in pediatric rheumatology has been the recognition that treatment response can be staged based on consensus criteria developed by an international panel [4], and that these stages have biological validity that can be characterized at the molecular level by gene transcriptional profiling [5-7]. Wallace *et al. *[4] defined these specific states as: active disease (AD), inactive disease (ID), clinical remission on medication (CRM), and clinical remission (CR). While true remission (CR) appears to be difficult to achieve (in the Wallace study [7], only 5% of children with the multiple-joint, polyarticular form of JIA achieved remission within 5 years of diagnosis), sustained periods of disease control (CRM) have become a reality and the target end point for childhood arthritis clinical trials. However, although achieving CRM has become commonplace in pediatric rheumatology clinical care, preliminary studies have suggested that the CRM biological state is not a return to normal, but, rather, a homeostatic state where pro-inflammatory disease networks are counterbalanced by the emergence of anti-inflammatory networks [6]. Indeed, peripheral blood gene expression abnormalities persist even in children who have been disease-free and off medication for a year or more [5,6].

Now that remission (or at least CRM) has become both the gold standard for clinical care and the end point for clinical trials for children, it is critical that we understand it at the molecular/biological level. One complication in doing so is that, while approximately 35 to 50% of children with JIA will experience CRM with the use of methotrexate (MTX) (usually in combination with nonsteroidal anti-inflammatory drugs +/- corticosteroids, used systemically or via joint injection), others will attain this state only after a biological agent, most commonly a TNF inhibitor, is added to methotrexate and the other agents [8]. However, whether the state of remission induced by MTX is, at the molecular level, identical to remission induced by the addition of a TNF inhibitor remains unknown, even though the remission phenotype is identical in each case. Answering this question is critical to our understanding of both the biology of response to therapy in JIA and toward our understanding the disease process itself. Furthermore, while there may be fundamental differences in the biology of response in adults compared to children and between the different disease entities in which anti-TNF therapies are used, the frequency with which remission (as defined here) can be achieved in children provides an excellent opportunity to understand mechanisms of response in such a way that these therapies might be manipulated in adults or in other diseases to achieve the same ends. Thus, understanding remission at the molecular level in this specific disease can be expected to have a useful impact on the multiple other chronic forms of arthritis in which immunosuppressive and anti-TNF therapies are used.

In this study, we used gene expression profiling to compare two groups of children with JIA who had achieved remission (CRM) to examine the medication-specific effects on gene transcriptional profiles.

## Materials and methods

### Patients and controls

This study was approved by the Oklahoma University Health Sciences Center (OUHSC) Institutional Review Board, and informed consent was obtained from all patients, or their parent/guardian, prior to the initiation of the study. We studied two independent cohorts of patients. One cohort was designated the training cohort, and the second, termed the testing cohort, was used to corroborate the results from the training cohort using quantitative, real-time PCR (qRT-PCR).

Children with polyarticular onset, rheumatoid factor (RF)-negative JIA were recruited from the OU Children's Physicians' rheumatology clinics and fit criteria for this subtype as specified by the International League of Associations for Rheumatology (ILAR) [9]. All children were on treatment at the time they were studied, and all fit criteria for CRM as defined by Wallace and colleagues [7]. That is, these children had all reached the ID state (normal physical examinations, absence of morning stiffness, and normal complete blood counts and erythrocyte sedimentation rates on laboratory monitoring studies) and, to fit criteria for CRM status, had maintained the ID state for 6 continuous months. The patients were followed every 2 to 3 months following their achieving ID, and CRM state samples were taken 6 to 8 months following the achievement of ID status. In the training cohort (for microarray), 14 children (ages 8.9 ± 3.1 years; seven females and seven males) achieved CRM with the use of MTX alone, 7 to 48 months after starting therapy. The patient comparison group in this cohort consisted of 14 other children with polyarticular JIA (ages 8.9 ± 4.2 years; 13 females and one male) who achieved CRM only after the addition of the TNF inhibitor, etanercept (Et), 11 to 48 months after the initiation of therapy. Both of these groups were compared to a group of 15 healthy children (ages 11.5 ± 2.6 years; seven female and eight male) recruited from the OU Children's Physicians' General Pediatrics clinic (Table 1).

**Table 1:** Demographic characteristics of study participants

Training group							
**Response to methotrexate**			**Response to methotrexate + etanercept**			**Healthy control**	

**Patients**	**Age**	**Time to CRM**	**Patients**	**Age**	**Time to CRM**	**Control**	**Age**
		**(month)**		**(month)**			

1	9	7	1	13	20	1	16
2	16	8	2	3	24	2	18
3*	11	10	3	3	48	3	9
4	7	14	4	6	8	4	11
5	11	12	5	4	18	5	10
6*	6	15	6	5	42	6	11
7	7	32	7	14	15	7	10
8*	11	13	8	9	25	8	10
9	7	36	9	10	30	9	11
10	4	46	10	15	25	10	13
11	11	8	11	11	20	11	13
12	7	15	12	12	24	12	10
13	11	48	13	12	24	13	12
14*	6	9	14	7	11	14	12

**Testing group**							

**Response to methotrexate**			**Response to methotrexate + etanercept**			**Healthy control**	

**Patients**	**Age**	**Time to CRM**	**Patients**	**Age**	**Time to CRM**	**Control**	**Age**
	**(month)**				**(month)**		

1	12	27	1	9	24	1	12
2	5	12	2	17	10	2	15
3	12	11	3	6	17	3	8
4	14	11	4	7	26	4	9
5	16	11	5	12	26	5	8
6	5	13	6	5	18	6	11
7	4	10	7	6	24	7	15
8	7	17	8	15	16	8	11

*RNA from granulocytes was insufficient for microarray analysis. CRM, clinical remission on medication.

A separate testing patient cohort of children with JIA was used to validate results from the first patient cohort. Eight of these children (ages 9.4 ± 4.7 years; eight females) achieved CRM with the use of MTX alone, 10 to 27 months from the initiation of therapy, and an additional eight children with polyarticular JIA (age 9.6 ± 4.5 years; eight females) achieved CRM only after the addition of Et, 10 to 26 months after the initiation of therapy. Eight healthy children (age 11.1 ± 2.8 years; four females and four males) were used as an independent comparison group to the testing cohort. Patient groups and characteristics are summarized in Table 1. All patients were treated with naproxen, 10 mg/kg/dose, as an adjunct to their primary drugs (that is, MTX +/- Et).

### Cell isolation

Whole blood was drawn into 10 mL citrated Cell Preparation Tubes (Becton Dickinson, Franklin Lakes, NJ, USA). Cell separation procedures were started within one hour from the time the specimens were drawn. Peripheral blood mononuclear cells (PBMC) were separated from granulocytes and red blood cells by density-gradient centrifugation. Red cells were removed from granulocytes by hypotonic lysis, and PBMC and granulocytes were then immediately placed in TRIzol^™^ reagent (Invitrogen, Carlsbad, CA, USA) and stored at -80°C.

### RNA isolation, labeling and gene expression profiling

Total RNA was extracted using TRIzol^™^ reagent according to the manufacturer's directions. RNA was further purified using a RNeasy MiniElute cleanup kit including a DNase digest according to the manufacturer's instructions (Qiagen, Valencia, CA, USA). RNA was quantified spectrophotometrically (Nanodrop, Thermo Fisher Scientific, Wilmington, DE, USA) and assessed for quality by capillary gel electrophoresis (Agilent 2100 Bioanalyzer; Agilent Technologies, Inc., Palo Alto, CA, USA). For the training cohort, sufficient amounts of high quality RNA for use in microarrays were obtained from 43 PBMC samples obtained from 14 JIA patients treated with MTX + Et, 14 JIA patients treated with MTX alone, and 15 healthy control children. From granulocytes, a sufficient amount of high quality RNA was obtained from 12 JIA patients treated with MTX + Et, 10 JIA patients treated with MTX alone, and 13 healthy control children. RNA samples were processed using GeneChip 3' IVT Express kit and hybridized to human U133 Plus 2.0 GeneChip^™^ microarrays according to the manufacturer's protocol (Affymetrix, Santa Clara, CA, USA). GeneChips^™^ were washed and stained using an Affymetrix automated GeneChip^™^ 450 fluidics station and scanned with an Affymetrix 3000 7G scanner. All gene expression data has been made available publically via the Gene Expression Omnibus (accession GSE41831).

### Statistical analysis and network modeling

CEL files were generated from scanned images using GeneChip^™^ Operating Software (GCOS, Affymetrix, version 1.3.0.037). Signal intensities were generated using JustRMA software (BRB-Array Tools). A log base 2 transformation was applied before the data were quantile normalized. Signal intensities were filtered using a log intensity variation (BRB-Array Tools) to obtain probes with the 25% highest variance across the arrays (13,668 probes) for further evaluation. Samples were divided into three groups (controls, patients treated with MTX + Et, and patients treated with MTX alone) and gene expression differences were separately evaluated in each patient group relative to controls. Differences between groups were considered statistically significant using a two-sample *t *test with univariate random variance model if the *P *value was ≤0.001 (BRB Array Tools, version 3.8.0 stable release). Statistically significant differentially expressed genes were filtered to obtain those with a minimal 1.3-fold change between groups and a mean expression level above background in at least one group. Annotations for probes were obtained from Affymetrix and were further supplemented by SOURCE [10]. These gene annotations were compared with the Gene Ontology (GO) database [11] to identify overrepresented terms using the R package GO available from Bioconductor within BRB-Array Tools [12]. A minimum of five observations in a GO class and parent class plus a minimum ratio of 2 for the observed vs. expected numbers were required for further consideration.

Interactions among differentially expressed genes in PBMC and granulocytes were analyzed using Ingenuity Pathway Analysis (IPA) software (Ingenuity Systems, Inc, Redwood City, CA, USA). Differentially expressed genes were mapped onto a global molecular network developed from information contained in the Ingenuity Pathways Knowledge Base.

### Gene expression validation by quantitative real-time RT-PCR

Total RNA (described above) was reverse transcribed with iScript^™^ cDNA synthesis kit according to the directions of the manufacturer (Bio-Rad, Hercules, CA, USA). Real-time RT-PCR was performed using SYBR Green reagents on an ABI Prism 7000 (for the training group; Applied Biosystems, Foster City, CA, USA) or a StepOne Plus (for the testing group; Applied Biosystems, Foster City, CA, USA). The temperature profile consisted of an initial 95°C step for 10 min, followed by 40 cycles of 95°C for 15 sec, 60°C for 1 min, and then a final melting curve analysis with a ramp from 60°C to 95°C over 20 min. Gene-specific amplification was confirmed by a single peak in the ABI Dissociation Curve software. Average threshold cycle (Ct) values for GAPDH (run in parallel reactions to the genes of interest) were used to normalize average Ct values of the gene of interest. These values were used to calculate averages for each group (healthy control or patient subsets), and the relative ΔCt was used to calculate fold-change values between the groups [5]. The nucleotide sequences of the primers are listed in Table 2.

**Table 2:** Primers used for quantitative real-time PCR validation.

Gene symbol	Primer direction^1^	Primer sequence (5' ~ 3')
BIRC3	F	CATGGGTTCAACATGCCAAGTGGT
BIRC3	R	TTCATCTCCTGGGCTGTCTGATGT
C3	F	GTGGAAATCCGAGCCGTTCTCT
C3	R	GATGGTTACGGTCTGCTGGTGA
CCR2	F	AGTTCAGAAGGTATCTCTCGGTG
CCR2	R	GGCGTGTTTGTTGAAGTCACT
CCR6	F	CTGAACCCTGTGCTCTACGCTT
CCR6	R	CACAGGAGAAGCCTGAGGACTT
CD22	F	GCGCAGCTTGTAATAGTTGGTGC
CD22	R	CACATTGGAGGCTGACCGAGTT
CXCR6	F	CAGTTCAGCAAGGTCTTTCTGCC
CXCR6	R	AGGTTCACCAGGAACACATCCG
EFR3A	F	ATTCAGTGCCATGTGCCATTCCTG
EFR3A	R	GCCCGAAGTTCATCGTTGACTGTT
FOXO1	F	CTACGAGTGGATGGTCAAGAGC
FOXO1	R	CCAGTTCCTTCATTCTGCACACG
FUZ	F	AGCCAGTTGTATCCACAGCTTCTG
FUZ	R	CCGAGGATGTCTGTGTGAAGGG
GM2A	F	TCGTTCCTGGAAATGTGACCCTCA
GM2A	R	CAGCTGCCAATGTAGTCTGTGCAT
GZMA	F	CCACACGCGAAGGTGACCTTAA
GZMA	R	CCTGCAACTTGGCACATGGTTC
IER5	F	ACCTCATCAGCATCTTCGGTTCCA
IER5	R	TTCATGTCTCTCAGCACCGGCTTA
IL6ST	F	CACCCTGTATCACAGACTGGCA
IL6ST	R	TTCAGGGCTTCCTGGTCCATCA
KLRD1	F	GAGCCAGCATTTACTCCAGGAC
KLRD1	R	GCACAGAGATGCCGACTTTCGT
KMO	F	ATACTCGAGTGGCTACCTTCACAC
KMO	R	TCTGATCTTCCAGGCCAACAGCTT
NBN	F	TCTGTCAGGACGGCAGGAAAGA
NBN	R	CACCTCCAAAGACAACTGCGGA
NSMAF	F	GTCTGAACACCTTCACGAGTGG
NSMAF	R	CTGTTCAAGTCTACACCTCCTTC
RNF167	F	ATGGGTCAGTCTTTATTGCGCTGC
RNF167	R	ATCCAGCCTTCTGGGCATTTAGGA
STAT1	F	ATGGCAGTCTGGCGGCTGAATT
STAT1	R	CCAAACCAGGCTGGCACAATTG
TARP	F	CCCCAAGCCCACTATTTTTC
TARP	R	TGTTGCTCTTCTTTTCTTGCC
TNFAIP6	F	TCACCTACGCAGAAGCTAAGGC
TNFAIP6	R	TCCAACTCTGCCCTTAGCCATC
TRAF3	F	AGCAGAGGTTGTGCAGAGCAGTTA
TRAF3	R	TCATCGGAACCTGACTCTTGCAGT
TRGV5	F	TGACTCAGGAAGACCAGCTC
TRGV5	R	TCTTAAAACTCCGGCCCCAC
TRIM4	F	ACGCCACACAGTGGAAGGATAAGA
TRIM4	R	TCTTCAACCAGGAAGTTGTGCAGC
TST	F	TTCCAGCTGGTGGATTCAAGGTCT
TST	R	AGAGATCCACCTTCTTGGTCTGGA
UBTF	F	ATGGATTCATAAGGCCCTGGAGCA
UBTF	R	TTTGTCCGAGAGCTGAGACCACT
XIAP	F	TGGCAGATTATGAAGCACGGATC
XIAP	R	AGTTAGCCCTCCTCCACAGTGA
ZNF277	F	AATGCAGGAAGACCGTGATGGGA
ZNF277	R	AGCGGCTCCAGGATACAATCCTTA

^1^Forward primer direction (F), reverse primer direction (R).

### Confocal microscopy for hepatocyte nuclear factor 4 alpha (HNF4α)

Human PBMC and granulocytes, isolated as described above, were adhered to poly-L-lysine-coated coverslips (Sigma-Aldrich, St. Louis, MO, USA) for 30 min at room temperature. Cells were fixed with 3% formaldehyde in PBS for 10 min at room temperature. After fixation, cells were treated with 0.25% Triton X-100 in PBS for 15 min. Slides were washed twice in PBS and blocked with blocking buffer (1% BSA, 1% donkey serum, 0.3M glycine in PBS containing 0.05% Tween 20) for 30 min. PBMC were incubated with rabbit anti-human HNF4α mAb (Cell Signaling Technology, Inc., Danvers, MA, USA) plus mouse anti-human CD4 mAb (Biolegend, San Diego, CA, USA), or rabbit anti-human HNF4α mAb plus mouse anti-human CD8 mAb (Biolegend, San Diego, CA, USA), and granulocytes were incubated with rabbit anti-human HNF4α mAb plus FITC-conjugated mouse anti-human CD66b mAb (BD Biosciences, San Jose, CA, USA), overnight at 4°C. After being washed with PBS, cells were incubated with Alexa Fluor 568- or 647-conjugated secondary antibody for 45 min at room temperature. After three rinses in PBS, the coverslips were mounted onto glass slides using ProLong Gold antifade reagent (Invitrogen, Carlsbad, CA, USA) with 4',6-diamidino-2-phenylindole (Invitrogen, Carlsbad, CA, USA). Fluorescent confocal laser scanning microscopy was conducted with a Leica SP2 MP laser scanning confocal microscope using the Leica confocal software LCS Lite (Leica Microsystems HD GmbH, Mannheim, Baden-Württemberg, Germany).

## Results

Our primary aim in this study was to determine whether the CRM state as achieved in a typical clinical setting results in a return to normal immune homeostasis in peripheral blood leukocytes. Preliminary studies from our research group [5,6] indicated that this was not likely, and this study, performed on a larger group of patients with independent corroboration in a second group of patients, was designed to answer that question in a definitive way and elucidate differences at the molecular level. To do this, we first compared each of the CRM groups (that is, MTX or MTX + Et) to healthy control children.

In both PBMC and in granulocytes, there were differences between children who achieved remission on MTX compared with those who achieved remission on MTX + Et relative to healthy control children. That is, although remission (CRM) is a distinct biological state, and phenotypically is indistinguishable among the groups, there were still differences in patterns of gene expression between and among the groups. For both cell types, hierarchical clustering of samples from the three groups (that is, healthy controls, children who had achieved remission on MTX, and children who had achieved remission on MTX + Et) revealed two clusters, each containing a similar proportion of samples from children in remission on MTX and healthy control children, while all but one of the samples from children who achieved remission on MTX + Et were grouped in one cluster. A 3 × 2 contingency table of these distributions revealed a nonrandom distribution of samples in both cell types (Table 3; PBMC: X^2 ^= 9.86, *P *<0.007; granulocytes: X^2 ^= 11.5, *P *<0.003). These results suggest that combined treatment with MTX + Et produced distinct gene expression responses that are distinct and more biologically focused at the gene expression level from the more heterogeneous responses detected among the MTX-treated patients, consistent with the idea that Et represents a more targeted therapy.

**Table 3:** Summary of hierarchical cluster analysis in PBMC and granulocytes from healthy control and in PBMC and granulocytes from children with JIA who achieved remission with methotrexate or methotrexate and etanercept

Cells	Group	Left Cluster	Right Cluster
PBMC	MTX + Et	1	14
	MTX	8	6
	Controls	8	7

Granulocytes	MTX + Et	13	1
	MTX	3	7
	Controls	5	7

ET, etanercept; JIA, juvenile idiopathic arthritis; MTX,

methotrexate; PBMC, peripheral blood mononuclear cells.

### Differences in PBMC gene expression profiles

Gene expression differences were detected in 67 genes represented by 75 probes when PBMC from JIA patients who achieved remission using MTX alone were compared to healthy controls. Twenty-two of these genes showed higher levels of expression and 45 showed lower levels in patient compared with control samples (Table S1 in Additional file 1). Thus, MTX appears to act not merely by suppressing pro-inflammatory genes, but by a re-ordering of specific transcript levels. Not surprisingly, functional associations among the differentially expressed genes included genes whose products are active in cell-mediated immunity. This included decreased expression of signal transducer and activator of transcription 1 (*STAT1*), which plays an important role mediating the effects of interferon gamma and in TH17 cell differentiation. STAT1 is activated by IL-6, an important cytokine in the pathogenesis of JIA [13] and IL-6 is known to be modulated by methotrexate [14,15]. Similarly, the expression of complement factor B, whose expression is increased by pro-inflammatory cytokines, was decreased in MTX-responsive patient samples. The decreased expression patterns of chemokine receptor 6, granzymes A and K, and the killer cell lectin like receptor subfamily D member 1 and subfamily K member 1 transcripts suggest modulation by MTX in cells of the innate (natural killer cells) and adaptive (cytotoxic T cells) immune systems in JIA. Input of the differentially expressed genes from this analysis into IPA software revealed a statistically significant downregulation of leukocyte activation in samples from the methotrexate-responsive samples based on the downregulation of five genes: *GZMA*, which activates monocytes [16], *STAT1 *whose phosphorylation leads to macrophage activation [17], *CALR*, which increases activation of dendritic cells [18], *SERPINB9*, which increases leukocyte activation [19], and *KLRK1*, which increases NK cell activation [20]. Upregulation of *CALR*, *PRDM1*, *STAT1*, *TAGAP *and *TNRC6B *has been associated elsewhere with adult rheumatoid arthritis or juvenile polyarticular arthritis [21-24]. Their downregulation here is consistent with an immunosuppressive effect of MTX across a variety of different molecules. Downregulation of *GZMA *by MTX was previously reported by Belinsky *et al. *[25].

Fifty-two genes represented by 56 different probe sets were differentially expressed in PBMC samples from patients treated with MTX + Et relative to samples from healthy controls (Table S2 in Additional file 2). Transcripts for 25 of these genes were expressed in higher levels in patients and 27 were expressed in lower levels compared with controls. GO analysis indicated an overrepresentation of products of the differentially expressed genes with roles in immunity, as expected. Of the 24 GO biological process categories obtained, 11 were related to immunity with ratios of the number of observed to expected genes varying from 4.57 to 22.05. The second-most overrepresented categories were related to histone or chromatin modification, with observed/expected ratios between 3.29 and 12.53. This finding is consistent with the hypothesis that alterations in gene expression that characterize the transition from active disease to remission may be accomplished alterations in chromatin accessibility through epigenetic alterations.

Twenty-one of the transcripts which showed differential expression when the MTX + Et group was compared with controls, including *KLRD1 *and *CFB*, were also differentially expressed in the MTX alone group vs. controls, suggesting either MTX-induced changes in the expression of these genes or persistence of pre-existing expression abnormalities not corrected by either drug regimen. *AGRN *and *KLRD1*, which showed decreased expression in the patient samples, are involved in T cell activation. CFB, AGRN and KLRD1 are involved in inflammatory response, while CFB and AGRN plus PPP1R14A are involved in cell-cell interactions. The remaining 31 genes were uniquely differentially expressed in the MTX + Et vs. controls comparison (including *CD22*, *CCR6 *and *TREM1 *which are of immunologic interest and suggest a unique effect of Et or MTX + Et interaction) and 46 that were uniquely differentially expressed in the MTX vs. controls (including *CXCR6*, *GZMA*, *TCRGC2*, *TCRGV5*, *TCR delta *and *STAT1*, which are of immunologic interest and suggest the unique effect of MTX). The increased expression of *CD22 *and decreased expression of *TREM1 *which occurred in patients who responded to the combined methotrexate and etanercept therapy but not those who responded to methotrexate alone suggests an Et-dependent effect on these molecules. *CD22 *is known to negatively regulate B cell activation [26,27], while *TREM1 *activation in monocytes induces pro-inflammatory cytokines and chemokines such as TNF, IL-1 and IL-6 [28,29]. The transcriptional modulation of these molecules in response to these therapies should reduce inflammation and modulate immune responses in responding patients.

#### Differential expression of genes in granulocytes

The most striking differences in gene expression profiles of patients with JIA responding to therapy were detected in granulocytes. A total of 207 differentially expressed genes were identified when samples from patients treated with MTX + Et were compared to control samples (Table S3 in Additional file 3). This contrasts with 23 genes that were differentially expressed in patients who achieved remission on MTX relative to controls (Table S4 in Additional file 4). That is, patients achieving remission on MTX alone had granulocyte gene expression profiles that more closely resembled normal than patients treated with MTX + Et. Four genes were upregulated in samples from patients that achieved remission with either therapeutic regimen, namely chromodomain helicase DNA-binding protein 2 (*CHD2*), RNA-binding motif protein 25 (*RBM25*), tripartite motif-containing 23 (*TRIM23*) and the *KIAA0907 *gene; seven genes were downregulated in both groups of patient samples relative to controls, namely forkhead box O1 (*FOXO1*), 3-phosphoinositide-dependent protein kinase-1 (*PDPK1*), PHD finger protein 20 (*PHF20*), splicing factor, arginine/serine-rich 18 (*SFRS18*), SAPS domain family, member 3 (*SAPS3*), neutral sphingomyelinase activation associated factor (*NSMAF*), and transmembrane protein 140 (*TMEM140*). FOXO proteins have been shown to regulate the expression of the TFN-related apoptosis-inducing ligand (*TRAIL*) [30], a TNF family member that can accelerate the rate of apoptosis in neutrophils [31-33]. TNF has been shown to induce granulocyte apoptosis in a dose-dependent manner, and via differential effects on expression of Mcl-1 and Bfl-1 [34-36]. Twenty-eight statistically significant (*P *<10^-2^) canonical pathways were identified from the MTX + Et differentially expressed genes using IPA, many of which are directly associated with inflammation, immunity, or apoptosis (Table 4). These include the RANKL signaling pathway, which regulates bone remodeling, the caspases-dependent apoptotic TWEAK signaling pathway, the CD27 apoptotic signaling pathway, the TNFR2 signaling pathway, the antigen-presenting CD40 signaling pathway, the TNF family and immunoregulatory APRIL and BAFF signaling pathways, the glucocorticoid receptor signaling pathway, and the IL-6 signaling pathway. Many of these differentially expressed genes, such as *ATM*, *BIRC3*, *MAP2K7*, *NFKBIE*, *TRAF3 *and *XIAP*, occurred in more than one signaling pathway. Based on the expression patterns of the molecules in Table 4 IPA software predicted decreased cellular apoptosis (3.24 × 10^-4^). Collectively, these findings demonstrate an important role of Et in the modulation of the innate immune system of responding patients, which may have implications for this and other diseases.

#### Between-group comparisons

Having established that CRM is not a return to normal, we next sought to determine the degree to which the homeostatic state induced by MTX alone resembled that induced by the combination of MTX + Et.

We first examined PBMC, comparing the gene expression profiles in JIA patients in remission following treatment with combined MTX + Et to samples from patients treated with MTX alone. Six genes were identified as being differentially expressed in PBMC between these groups (Table S5 in Additional file 5). Four genes were upregulated in patients treated with the combined therapy: cardiotrophin-like cytokine factor 1 *CLCF1 *complement component 3 (*C3*), nonprotein-encoding XIST antisense RNA (*TSIX*), and one gene currently lacking annotation (Affymetrix probe set ID 240861_at). Two genes were downregulated in patients using combined therapy: insulin-like growth factor 1 receptor (IGF1R) and the Y-linked protein kinase gene (PRKY).

When the granulocyte expression profiles of children who achieved remission on MTX were compared directly with those who achieved remission on MTX + Et, we found 33 genes (42 probes) that showed differences in expression. This was an expected finding, given that children who had remission on MTX alone had expression profiles that more closely resembled the healthy controls than did children who achieved remission on MTX + Et.

Differential gene expression was also detected with three different probes for the eukaryotic translation initiation factor 1A, Y-linked gene (*EIF1AY*) with no evidence of expression in the MTX + Et samples relative to the MTX alone samples, with six different probes for the nonprotein-coding × (inactive)-specific transcript *(XIST) *with expression above background only in the MTX + Et-treated samples, and with three different probes for the nibrin gene (NBN) expressed above background signal intensities in both groups. Only one gene, the insulin-like growth factor 1 receptor gene (IGF1R), was differentially expressed in both PBMC and neutrophils. However, this gene was overexpressed in granulocytes from patients treated with MTX + Et, but underexpressed in MTX + Et-treated PBMC samples. Collectively, these findings indicate very different effects of Et on different subsets of peripheral blood.

The nibrin gene was overexpressed in MTX + Et relative to both MTX only and controls, as were the genes encoding for cytochrome b-245, beta polypeptide (*CYBB*), haloacid dehalogenase-like hydrolase domain-containing 1A (*HDHD1A*), baculoviral IAP repeat-containing 3 (BIRC3), TNF receptor-associated factor 3 (*TRAF3*), and × (inactive)-specific transcript (*XIST*). The histone cluster 1, H1c (HIST1H1C) gene was found to be downregulated in MTX + Et relative to both MTX only and control samples. No overlap was found among the differentially expressed genes in the MTX only vs. MTX + Et and the MTX only vs. control samples (Table S6 in Additional file 6).

#### Network analysis of differentially expressed genes

**Figure 1:**
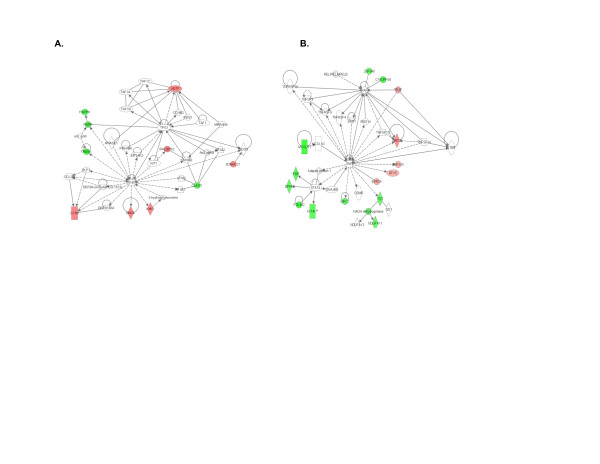
**Networks derived from Ingenuity Pathway Analysis software of children who achieved CRM status using MTX + Et compared with healthy controls**. Network analysis in PBMC is shown in **(A) **and analysis in granulocytes is shown in **(B)**. Note prominence of TNFA as a hub in these networks. Genes shown in red show higher expression in patients compared with controls, and those shown in green show lower expression..CRM, clinical remission on medication; ET, etanercept; MTX, methotrexate; PBMC, peripheral blood mononuclear cells.

**Figure 2:**
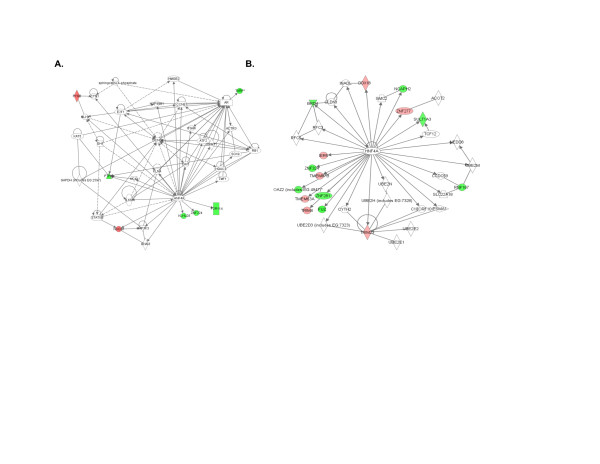
**Network derived from Ingenuity Pathway Analysis software of children who achieved CRM status using MTX + Et **(A) **or MTX alone **(B) **compared with healthy controls**. Network analysis is from PBMC (A) and granulocytes (B). Note the prominence of HNF4α as a hub in both networks. Genes shown in red show higher expression in patients compared with controls, and those shown in green show lower expression. CRM, clinical remission on medication; ET, etanercept; MTX, methotrexate; PBMC, peripheral blood mononuclear cells.

Functional associations between differentially expressed genes identified above were analyzed using the IPA software. It is interesting to note that many of these networks contained hub-and-node structures characteristic of scale-free systems [37] as we have previously reported [38]. While some of these structures may be artifacts that emerge from the algorithms used by IPA to query the existing literature, there is biological coherence in many of the networks, all of which were generated in an unbiased fashion. For example, in both PBMC (Figure 1A, and Figure S1 in Additional file 7) and granulocytes (Figure 1B, and Figure S2 in Additional file 8), TNF alpha appears as a hub in at least one network, as would be predicted given Et's mechanism of action. We also noted hub-and-node structured networks derived from both types of cells that demonstrated interactions between the steroid hormone receptor/transcription factor hepatocyte nuclear factor 4 alpha (HNF4α) and differentially expressed gene products in both types of cells (Figure 2). Connections in these networks reflect HNF4α binding to DNA sequences in or adjacent to these genes that were identified by chromatin immunoprecipitation assays [39]. Because HNF4α had not been reported to be expressed in leukocytes, we undertook experiments to investigate this finding further.

#### Expression of HNF4α in leukocytes

**Figure 3:**
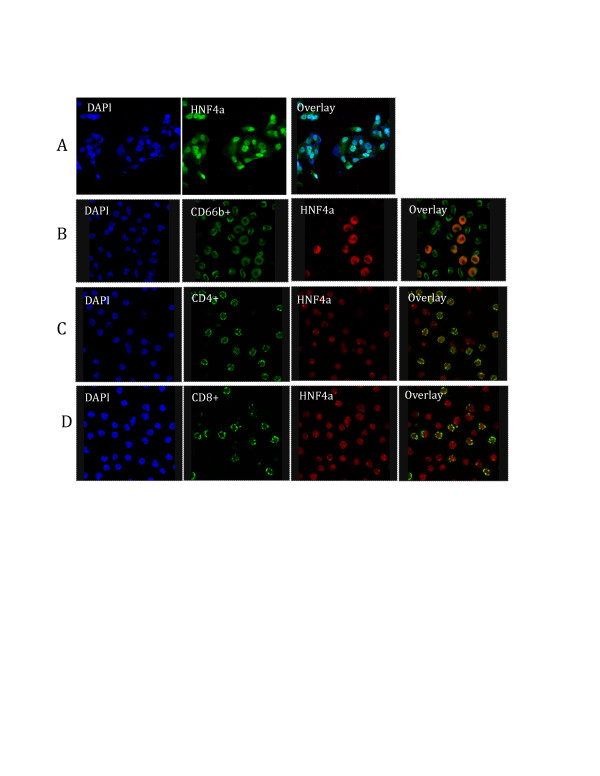
**Expression of HNF4α in leukocytes**. Indirect immunofluorescence microscopy was used to detect the expression of HNF4α. Cells were fixed in 4% paraformaldehyde and stained with antibodies against human CD4, CD8, CD66b, and HNF4α. **(A) **HepG2 cells as positive control. **(B) **Granulocytes. **(C **and **D) **Peripheral blood mononuclear cells.

Network analyses of the microarray data suggested a role for HNF4α in regulating a number of genes associated with remission (for example, Figure 2**)**. HNF4α is a transcription factor and a steroid hormone receptor superfamily member that is expressed mainly in liver and kidney, and at lower levels in pancreatic islets, small intestine and colon [40]. Given the relationship between HNF4α and a number of the differentially expressed genes in PBMC and granulocytes, we tested for the presence of HNF4α protein in human leukocytes using immunofluorescence microscopy. As a positive control, we observed intense nuclear and light cytoplasmic staining in human hepatocellular carcinoma HepG2 cells (Figure 3A). We next examined CD66b+ granulocytes, CD4+ T cells, and CD8+ T cells and detected HNF4α in each of these leukocyte subsets. In T cells, HNF4α immunofluorescent signals were of similar intensity in CD8+ and in CD4+ T cells and lower than intensities observed in CD66b+ granulocytes. All T cells were immunofluorescent positive for HNF4α while one-third of CD66b+ cells were positive. Staining in CD66b+ cells was primarily cytoplasmic, while nuclear and cytoplasmic staining was observed among each T cell subpopulation. All of these findings were observed in cells from healthy children, healthy adults, and children with JIA (data not shown). The findings support the hypothesis that HNF4α is expressed in each of these types of cells and indirectly corroborate the functional interaction of gene products in the networks reported above.

#### Validation of microarray data

**Figure 4:**
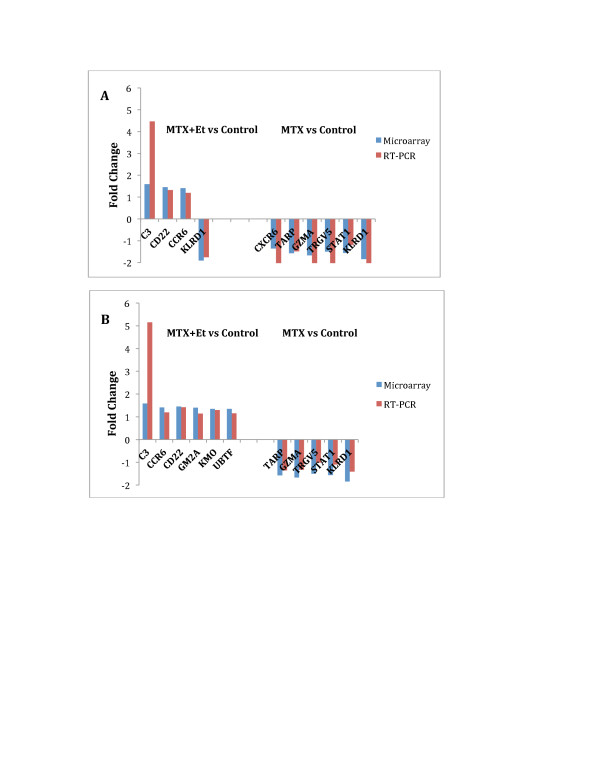
**Validation of microarray data for selected genes by real-time PCR**. **(A **and **B) **Differentially expressed genes in PBMC from JIA patients who achieved remission using MTX alone or MTX + Et were compared to healthy controls in training cohort (A) and testing cohort (B). (**C **and **D**) Differentially expressed genes in granulocytes from JIA patients who achieved remission using MTX alone or MTX + Et were compared to healthy controls in training cohort (C) and testing cohort (D). ET, etanercept; JIA, juvenile idiopathic arthritis; MTX, methotrexate; PBMC, peripheral blood mononuclear cells.

We performed quantitative real-time PCR on RNA obtained from granulocytes and PBMC from the training cohort of patients and healthy controls to confirm the altered pattern of gene expression detected with microarrays. Ten differentially expressed genes identified from microarray expression patterns of PBMC were evaluated (Figure 4A). All genes were similarly over- or under-expressed using both methods. In granulocytes, nine genes that were tested by qRT-PCR exhibited agreement between microarray and quantitative real-time PCR results (Figure 4C). To further confirm the findings from the microarray results, 12 differentially expressed genes identified from microarray expression patterns in PBMC and 14 differentially expressed genes identified from microarray expression patterns in granulocytes were evaluated by quantitative real-time PCR on RNA obtained from the independent testing cohort. The PCR results confirmed the differential expression of 11 of the 12 genes in PBMC (Figure 4B, 92% validation) and 13 of the 14 genes in granulocytes (Figure 4D, 93% validation). The differentially expressed gene *CXCR6 *in PBMC and *FOXO1 *in neutrophils were not been validated by PCR (data not shown). Five (*IER5*, *FUZ*, *RNF167*, *TRIM4 *and *ZNF277*) of the 13 genes we validated in granulocytes were also relevant directly to the HNF4a network mentioned above. *TARP*, which was present in the PBMC network with an *HNF4A *hub (above), was validated by PCR in both training and testing groups. Thus, PCR experiments, including those performed on an independent patient cohort, corroborated results from both the expression and network analysis data.

## Discussion

Significant advances have been made in the past 10 to 15 years in the treatment of JIA. Indeed, it is now possible to achieve remission in the majority of children with even the more severe polyarticular-onset forms of this disease, although sustained periods without clinical disease can currently be achieved and maintained only by sustained use of immunosuppressive medications. While preliminary studies from our group suggested that remission (both CRM and CR) in JIA represents a distinct biological 'state' that can be recognized at the molecular level as well as clinically [6], it is critical to derive a deeper understanding of the biological meaning of these states. In particular, since children with JIA can achieve remission on different medications, it is of great interest to know whether remission achieved on MTX leads to an identical immunologic/biologic state as that achieved on a TNF inhibitor. We demonstrate here that children who have achieved the CRM state show significant differences from healthy controls, although those differences are more pronounced in the group who achieved remission on MTX + Et compared with those who achieved remission on MTX alone. The findings here corroborate our smaller, preliminary studies [22] and demonstrate that the CRM state is not a return to 'normal', but rather a re-ordering of transcriptional profiles in leukocytes (and very possibly other cells or tissues) in such a way that pro-inflammatory responses are counterbalanced by anti-inflammatory responses. Furthermore, this re-ordering occurs in cells of both the innate and adaptive immune systems. Findings in this study are thus consistent with previously published work [5,6,22,40] suggesting that, rather than being driven purely by aberrant adaptive immune processes, the pathogenesis of polyarticular JIA likely involves complex interactions between innate and adaptive immunity. For example, 21 of the differentially expressed genes in PBMC in patients (MTX + Et and MTX alone) relative to controls are known to be involved in T cell activation (*AGRN *and *KLRD1*), inflammation response (CFB, AGRN and KLRD1), and cell-cell interaction (*CFB*, *AGRN *and *PPP1R14A*). The differentially expressed genes in PBMC in patients (MTX alone) relative to controls are involved in T cell and NK cell proliferation (*STAT1 *and *KLRK1*) and in T cell apoptosis and death (*SERPINB9*, *CALR*, *PRDM1 *and *GZMA*).

The more dramatic differences between children in remission and healthy control children were observed in the expression profiles of granulocytes. This was especially true when we compared children in remission on MTX + Et with the control population. In that comparison, there were 207 genes that showed differential expression. Not surprisingly, many of the differentially expressed genes act through TNF-associated pathways. For example, *ABCF1 *can be regulated by TNF-alpha and play a role in enhancement of protein synthesis and the inflammation process [41]. *TNFAIP6*, increased in JIA patient granulocytes, can be induced by pro-inflammatory cytokines such as TNF-alpha and IL-1 [42]. Enhanced levels of TNFAIP6 protein have also been found in the synovial fluid of patients with osteoarthritis and rheumatoid arthritis [43]. GCH1 protein expression and enzyme activity are strongly induced by a mixture of three pro-inflammatory cytokines, IL-1beta, TNF-alpha, and IFN-gamma [44]. XIAP belongs to a family of apoptotic suppressor proteins and acts by binding to TNF receptor-associated factors TRAF1 and TRAF2. This protein also inhibits at least two members of the caspase family of cell-death proteases, caspase-3 and caspase-7. XIAP also regulates innate immune responses by interacting with NOD1 and NOD2 through interaction with RIP2 [45,46].

Despite the large number of differentially expressed genes in granulocytes of patients who responded to MTX + Et therapy, only 23 differentially expressed genes in granulocytes were identified in patients that achieved remission on MTX relative to controls. This finding argues that the majority of the MTX +Et transcriptome changes were affected by Et. Nevertheless, there may be functional overlap in genes affected by these therapeutic regimens. For example, among genes affected by MTX, neutral sphingomyelinase (N-SMase) activation associated factor (*NSMAF*), downregulated in MTX-treated patient vs. control samples, is an adaptor protein that constitutively binds to TNF-R1 and is involved in TNF-induced gene expression such as IL-6 and CXCL-2, and leukocyte recruitment, contributing to the establishment of the specific immune response [47]. The effect of these drugs is not limited to TNF-related modifications. Eleven genes (*CHD2*, *KIAA0907*, *PHF20*, *RBM25*, *NSMAF*, *FOXO1*, *PDPK1*, *SAPS3*, *SFRS18*, *TMEM140 *and *TRIM23*) were differentially expressed following treatment with MTX or MTX + Et, and participate in various cellular processes including cellular development, carbohydrate metabolism, cell morphology, cell death and gene expression.

A number of differentially expressed genes identified in this study have been shown to bind the transcription factor HNF4α [40]. HNF4α belongs to the steroid hormone receptor superfamily and is enriched in liver [48]. HNF4α contributes to regulation of a large fraction of the liver and pancreatic islet transcriptomes by binding directly to nearly half of the actively transcribed genes in those tissues and plays a role in regulating the cytokine-induced inflammatory response [41,49]. Based on the predicted interaction between HNF4α and a number of differentially expressed genes in this study, we demonstrated expression of HNF4α in leukocytes at the protein level in PBMC and granulocytes from patients with JIA and from healthy controls using immunofluorescence assays. *HNF4A *transcripts were expressed above background signal intensities on the microarrays (data not shown). Our results support the hypothesis that HNF4α controls many genes associated with remission. Although we did not see significant differences in the expression levels of *HNF4A *transcripts in leukocytes between patients and healthy controls, HNF4α may be controlled by posttranscriptional events or may act as a cofactor, interacting with other transcription factors, for example ETS-domain transcription factor ELK1, and not directly bind to DNA to regulate these genes [50]. Some ETS family proteins interact with other transcription factors (AP-1, NF-κB and Stat-5) to co-regulate the expression of cell-type-specific genes, and these interactions coordinate cellular processes in response to diverse signals from cytokines, growth factors, antigens, and cellular stresses [51]. The differences in the intracellular localization of HNF4α observed here in granulocytes and T cells remain to be explained. They may be related to the abundance of other transcriptional binding factors in such cells or to mutations or exon splice variations that are present in the *HNF4A *gene.

Together, our results demonstrate that the remission state in JIA is not the result of a normalization of immune homeostasis. Gene expression in both PBMC and granulocytes remains abnormal when patients in remission are compared with healthy control children. Furthermore, while there are some overlapping points, remission achieved on MTX differs from remission achieved on MTX + Et, especially in granulocytes, suggesting overlapping but not identical 'set points' for each of these remission states.

These findings provide insight into one of the single most important clinical features of chronic arthritis in children: the frequency of disease recurrence and the rarity of true remission (defined by the Wallace group as a full year off all medications without recurrence of disease signs or symptoms). Our studies show that the CRM state is still associated with distinct differences between children in remission (who appear to be completely normal) and perfectly healthy children. The degree to which these abnormalities reflect persistence of the underlying condition itself or a new immunologic homeostasis that emerges because of the drugs themselves is unclear, although our earlier studies [5,6] strongly suggest the latter. Longitudinal studies will be required to monitor the expression of these or other genes prior to, throughout, and after treatment to identify biomarkers that may predict which patients with JIA are likely to respond to particular therapeutic regimens to optimize therapy in the future. These findings represent the first steps in the identification of such molecules.

## Conclusions

Remission (CRM) in polyarticular JIA is a distinct biological state that differs from normal. Gene expression profiles of PBMC show little differences whether the CRM state was achieved with MTX or the combination of MTX + Et. However, the CRM state in JIA neutrophils show significant differences depending on whether remission was achieved with MTX or MTX + Et. These findings therefore add evidence to the importance of neutrophils in the pathogenesis of JIA and its response to therapy.

## Abbreviations

AD: active disease; BSA: bovine serum albumin; CR: clinical remission; CRM: clinical remission on medication; ET: etanercept; GO: gene ontology; ID: inactive disease; IL: interleukin; IPA: Ingenuity Pathways Analysis; JIA: juvenile idiopathic arthritis; mAB: monoclonal antibody; MTX: methotrexate; PBS: phosphate-buffered saline; qRT-PCR: quantitative real-time reverse-transcription polymerase chain reaction; PBMC: peripheral blood mononuclear cells; TNF: tumor necrosis factor.

## Competing interests

The authors declare that they have no competing interests.

## Authors' contribution

KJ, BF and JJ designed and executed the study, analyzed data and prepared the manuscript. YC was involved in sample acquisition and RNA preparation. JO was involved in RNA labeling and scanning. All authors read and approved the final manuscript.

## Supplementary Material

Additional file 1 **Table S1**. Differentially expressed genes in PBMC in JIA patients who achieved remission with methotrexate alone vs. controls. Genes listed more than once indicate different probes for the same gene which showed different values in expression. Click here for file

Additional file 2 **Table S2**. Differentially expressed genes in PBMC in JIA patients who achieved remission with etanercept and methotrexate vs. controls. Genes listed more than once indicate different probes for the same gene which showed different values in expression. Click here for file

Additional file 3 **Table S3**. Differentially expressed genes in granulocytes of JIA patients who achieved remission with methotrexate and etanercept vs. controls. Genes listed more than once indicate different probes for the same gene which showed different values in expression. Click here for file

Additional file 4 **Table S4**. Differentially expressed genes in granulocytes of JIA patients who achieved remission with methotrexate alone vs. controls. Genes listed more than once indicate different probes for the same gene which showed different values in expression. Click here for file

Additional file 5 **Table S5**. Differentially expressed genes in PBMC in JIA patients who achieved remission with methotrexate and etanercept vs. methotrexate alone. Click here for file

Additional file 6 **Table S6**. Differentially expressed genes in granulocytes in JIA patients who achieved remission with methotrexate and etanercept vs. methotrexate alone. Genes listed more than once indicate different probes for the same gene which showed different values in expression. Click here for file

Additional file 7 **Figure S1**. Interactions between products of differentially expressed genes in PBMC from patients with JIA who achieved remission using methotrexate alone (A) or Etanercept and Methotrexate (B) relative to PBMC from controls. Differentially expressed genes entered in the Ingenuity Pathway Analysis program are colored. Genes shown in red show higher expression in patients compared with controls, and those shown in green show lower expression. Genes not colored were added by the IPA program to generate these networks. Click here for file

Additional file 8 **Figure S2**. Interactions between products of differentially expressed genes in granulocytes from patients with JIA who achieved remission using methotrexate alone (A) or Etanercept and Methotrexate (B) relative to granulocytes from controls. Differentially expressed genes entered in the Ingenuity Pathway Analysis program are colored. Genes shown in red show higher expression in patients compared with controls, and those shown in green show lower expression. Genes not colored were added by the IPA program to generate these networks. Click here for file

## References

[B1] Ringold S Wallace CA Measuring clinical response and remission in juvenile idiopathic arthritis Curr Opin Rheumatol 2007 15 471 476 10.1097/BOR.0b013e32825a6a68 17762613

[B2] Jarvis JN Juvenile rheumatoid arthritis: a guide for pediatricians Pediatr Ann 2002 15 437 446 10.3928/0090-4481-20020701-08 12149797

[B3] Shenoi S Wallace CA Remission in juvenile idiopathic arthritis: current facts Curr Rheumatol Reports 2010 15 80 86 10.1007/s11926-010-0085-2 20425015

[B4] Wallace CA Ruperto N Giannini E Childhood Arthritis and Rheumatology Research Alliance Pediatric Rheumatology International Trials Organization Pediatric Rheumatology Collaborative Study Group Preliminary criteria for clinical remission for select categories of juvenile idiopathic arthritis J Rheumatol 2004 15 2290 2294 15517647

[B5] Jarvis JN Jiang K Frank MB Knowlton N Aggarwal A Wallace CA McKee R Chaser B Tung C Smith LB McGhee JL Chen Y Osban J O'Neil KN Centola M Gene expression profiling in neutrophils of children with polyarticular juvenile idiopathic arthritis Arthritis Rheum 2009 15 1488 1495 10.1002/art.24450 19404961 PMC3063001

[B6] Knowlton N Jiang K Frank MB Aggarwal A Wallace C McKee R Chaser B Tung C Smith L Chen Y Osban J O'Neil K Centola M McGhee JL Jarvis JN The meaning of clinical remission in polyarticular juvenile idiopathic arthritis: gene expression profiling in peripheral blood mononuclear cells identifies distinct disease states Arthritis Rheum 2009 15 892 900 10.1002/art.24298 19248118 PMC2758237

[B7] Wallace CA Huang B Bandeira M Ravelli A Giannini EH Patterns of clinical remission in select categories of juvenile idiopathic arthritis Arthritis Rheum 2005 15 3354 3562 10.1002/art.21389 16255044

[B8] Shenoi S Wallace CA Tumor necrosis factor inhibitors in the management of juvenile idiopathic arthritis: an evidence-based review Paediatric Drugs 2010 15 367 377 10.2165/11532610-000000000-00000 21028916

[B9] Petty RE Southwood TR Manners P Baum J Glass DN Goldenberg J He X Maldonado-Cocco J Orozco-Alcala J Prieur AM Suarez-Almazor ME Woo P International League of Associations for Rheumatology classification of juvenile idiopathic arthritis: second revision Edmonton, 2001 J Rheumatol 2004 15 390 392 14760812

[B10] Diehn M Sherlock G Binkley G Jin H Matese JC Hernandez-Boussard T Rees CA Cherry JM Botstein D Brown PO Alizadeh AA SOURCE: a unified genomic resource of functional annotations, ontologies, and gene expression data Nucleic Acids Research 2003 15 219 223 10.1093/nar/gkg014 12519986 PMC165461

[B11] Ashburner M Ball CA Blake JA Botstein D Butler H Cherry JM Davis AP Dolinski K Dwight SS Eppig JT Harris MA Hill DP Issel-Tarver L Kasarskis A Lewis S Matese JC Richardson JE Ringwald M Rubin GM Sherlock G Gene ontology: tool for the unification of biology. The Gene Ontology Consortium Nature Genet 2000 15 25 29 10.1038/75556 10802651 PMC3037419

[B12] Gentleman RC Carey VJ Bates DM Bolstad B Dettling M Dudoit S Ellis B Gautier L Ge Y Gentry J Hornik K Hothorn T Huber W Iacus S Irizarry R Leisch F Li C Maechler M Rossini AJ Sawitzki G Smith C Smyth G Tierney L Yang JY Zhang J Bioconductor: open software development for computational biology and bioinformatics Genome Biology 2004 15 R80 10.1186/gb-2004-5-10-r80 15461798 PMC545600

[B13] Piekorz RP Nemetz C Hocke GM Members of the family of IL-6-type cytokines activate Stat5a in various cell types Biochem Biophys Res Commun 1997 15 438 443 10.1006/bbrc.1997.6976 9240457

[B14] Huang Q Jin X Gaillard ET Knight BL Pack FD Stoltz JH Jayadev S Blanchard KT Gene expression profiling reveals multiple toxicity endpoints induced by hepatotoxicants Mutat Res 2004 15 147 167 10.1016/j.mrfmmm.2003.12.020 15120968

[B15] Kim YJ Song M Ryu JC Inflammation in methotrexate-induced pulmonary toxicity occurs via the p38 MAPK pathway Toxicology 2009 15 183 190 10.1016/j.tox.2008.11.016 19100307

[B16] Sower LE Froelich CJ Allegretto N Rose PM Hanna WD Klimpel GR Extracellular activities of human granzyme A. Monocyte activation by granzyme A versus alpha-thrombin J Immunol 1996 15 2585 2590 8786323

[B17] Takeda K Akira S STAT family of transcription factors in cytokine-mediated biological responses Cytokine Growth Factor Rev 2000 15 199 207 10.1016/S1359-6101(00)00005-8 10817963

[B18] Proskuryakov SY Konoplyannikov AG Gabai VL Necrosis: a specific form of programmed cell death? Exp Cell Res 2003 15 1 16 10.1016/S0014-4827(02)00027-7 12565815

[B19] Lovo E Zhang M Wang L Ashton-Rickardt PG Serine protease inhibitor 6 is required to protect dendritic cells from the kiss of death J Immunol 2012 15 1057 1063 10.4049/jimmunol.1102667 22227570 PMC3270301

[B20] Sutherland CL Chalupny NJ Schooley K VandenBos T Kubin M Cosman D UL16-binding proteins, novel MHC class I-related proteins, bind to NKG2D and activate multiple signaling pathways in primary NK cells J Immunol 2002 15 671 679 10.4049/jimmunol.168.2.671 11777960

[B21] Andreakos E Sacre S Foxwell BM Feldmann M The toll-like receptor-nuclear factor kappaB pathway in rheumatoid arthritis Front Biosci 2005 15 2478 2488 10.2741/1712 15970510

[B22] Jarvis JN Petty HR Tang Y Frank MB Tessier PA Dozmorov I Jiang K Kindzelski A Chen Y Cadwell C Turner M Szodoray P McGhee JL Centola M Evidence for chronic peripheral activation of neutrophils in polyarticular juvenile rheumatoid arthritis Arthritis Res Ther 2006 15 R154 10.1186/ar2048 17002793 PMC1779452

[B23] Nakamura N Shimaoka Y Tougan T Onda H Okuzaki D Zhao H Fujimori A Yabuta N Nagamori I Tanigawa A Sato J Oda T Hayashida K Suzuki R Yukioka M Nojima H Ochi T Isolation and expression profiling of genes upregulated in bone marrow-derived mononuclear cells of rheumatoid arthritis patients DNA Res 2006 15 169 183 10.1093/dnares/dsl006 17082220

[B24] Goëb V Thomas-L'Otellier M Daveau R Charlionet R Fardellone P Le Loët X Tron F Gilbert D Vittecoq O Candidate autoantigens identified by mass spectrometry in early rheumatoid arthritis are chaperones and citrullinated glycolytic enzymes Arthritis Res Ther 2009 15 R38 10.1186/ar2644 19284558 PMC2688184

[B25] Belinsky GS Parke AL Huang Q Blanchard K Jayadev S Stoll R Rothe M Achenie LE Gupta RR Wu GY Rosenberg DW The contribution of methotrexate exposure and host factors on transcriptional variance in human liver Toxicol Sci 2007 15 582 594 10.1093/toxsci/kfm067 17400583

[B26] Cyster JG Goodnow CC Tuning antigen receptor signaling by CD22: integrating cues from antigens and the microenvironment Immunity 1997 15 509 517 10.1016/S1074-7613(00)80339-8 9175829

[B27] Nitschke L Carsetti R Ocker B Köhler G Lamers MC CD22 is a negative regulator of B-cell receptor signalling Curr Biol 1997 15 133 143 10.1016/S0960-9822(06)00057-1 9016707

[B28] Netea MG Azam T Ferwerda G Girardin SE Kim SH Dinarello CA Triggering receptor expressed on myeloid cells-1 (TREM-1) amplifies the signals induced by the NACHT-LRR (NLR) pattern recognition receptors J Leukoc Biol 2006 15 1454 1461 10.1189/jlb.1205758 16940328

[B29] Dower K Ellis DK Saraf K Jelinsky SA Lin LL Innate immune responses to TREM-1 activation: overlap, divergence, and positive and negative cross-talk with bacterial lipopolysaccharide J Immunol 2008 15 3520 3534 10.4049/jimmunol.180.5.3520 18292579

[B30] Modur V Nagarajan R Evers BM Milbrandt J FOXO proteins regulate tumor necrosis factor-related apoptosis inducing ligand expression. Implications for PTEN mutation in prostate cancer J Biol Chem 2002 15 47928 47937 10.1074/jbc.M207509200 12351634

[B31] Renshaw SA Parmar JS Singleton V Rowe SJ Dockrell DH Dower SK Bingle CD Chilvers ER Whyte MK Acceleration of human neutrophil apoptosis by TRAIL J Immunol 2003 15 1027 1033 10.4049/jimmunol.170.2.1027 12517970

[B32] Wright HL Moots RJ Bucknall RC Edwards SW Neutrophil function in inflammation and inflammatory diseases Rheumatology (Oxford) 2010 15 1618 1631 10.1093/rheumatology/keq045 20338884

[B33] Grivennikov SI Tumanov AV Liepinsh DJ Kruglov AA Marakusha BI Shakhov AN Murakami T Drutskaya LN Förster I Clausen BE Tessarollo L Ryffel B Kuprash DV Nedospasov SA Distinct and nonredundant in vivo functions of TNF produced by t cells and macrophages/neutrophils: protective and deleterious effects Immunity 2005 15 93 104 15664162 10.1016/j.immuni.2004.11.016

[B34] Salamone G Giordano M Trevani AS Gamberale R Vermeulen M Schettinni J Geffner JR Promotion of neutrophil apoptosis by TNF-alpha J Immunol 2001 166 3476 3483 11207306 10.4049/jimmunol.166.5.3476

[B35] van den Berg JM Weyer S Weening JJ Roos D Kuijpers TW Divergent effects of tumor necrosis factor alpha on apoptosis of human neutrophils J Leukoc Biol 2001 15 467 473 11261795

[B36] Cross A Moots RJ Edwards SW The dual effects of TNFalpha on neutrophil apoptosis are mediated via differential effects on expression of Mcl-1 and Bfl-1 Blood 2008 15 878 884 10.1182/blood-2007-05-087833 17942758

[B37] Barbasi AL Albert A Emergence of scaling in networks Science 1999 15 509 512 10.1126/science.286.5439.509 10521342

[B38] Jeong H Tombor B Oltval ZN Barabasi A-L The large-scale organization of metabolic networks Nature 2000 15 651 654 10.1038/35036627 11034217

[B39] Frank MB Wang S Aggarwal A Knowlton N Jiang K Chen Y McKee R Chaser B McGhee T Osban J Jarvis JN Disease-associated pathophysiologic structures in pediatric rheumatic diseases show characteristics of scale-free networks seen in physiologic systems: implications for pathogenesis and treatment BMC Med Genomics 2009 15 9 23 10.1186/1755-8794-2-9 19236715 PMC2649160

[B40] Odom DT Zizlsperger N Gordon DB Bell GW Rinaldi NJ Murray HL Volkert TL Schreiber J Rolfe PA Gifford DK Fraenkel E Bell GI Young RA Control of pancreas and liver gene expression by HNF transcription factors Science 2004 15 1378 1381 10.1126/science.1089769 14988562 PMC3012624

[B41] Ihara A Yamagata K Nammo T Miura A Yuan M Tanaka T Sladek FM Matsuzawa Y Miyagawa J Shimomura I Functional characterization of the HNF4alpha isoform (HNF4alpha8) expressed in pancreatic beta-cells Biochem Biophys Res Commun 2005 15 984 990 10.1016/j.bbrc.2005.02.072 15752752

[B42] Richard M Drouin R Beaulieu AD ABC50, a novel human ATP-binding cassette protein found in tumor necrosis factor-alpha-stimulated synoviocytes Genomics 1998 15 137 145 10.1006/geno.1998.5480 9790762

[B43] Wisniewski HG Hua JC Poppers DM Naime D VilEek J Cronsteint BN TNF/IL-1 -inducible protein TSG-6 potentiates plasmin inhibition by inter-alpha-inhibitor and exerts a strong anti-inflammatory effect in vivo J Immunol 1996 15 1609 1615 8568267

[B44] Fujikado N Saijo S Iwakura Y Identification of arthritis-related gene clusters by microarray analysis of two independent mouse models for rheumatoid arthritis Arthritis Res Ther 2006 15 R100 10.1186/ar1985 16805906 PMC1779393

[B45] Chiarini A Armato U Pacchiana R DalPra I Proteomic analysis of GTP cyclohydrolase 1 multiprotein complexes in cultured normal adult human astrocytes under both basal and cytokine-activated conditions Proteomics 2009 15 1850 1860 10.1002/pmic.200800561 19294699

[B46] Sanna MG da Silva Correia J Luo Y Chuang B Paulson LM Nguyen B Deveraux QL Ulevitch RJ ILPIP, a novel anti-apoptotic protein that enhances XIAP-mediated activation of JNK1 and protection against apoptosis J Biol Chem 2002 15 30454 30462 10.1074/jbc.M203312200 12048196

[B47] Krieg A Correa RG Garrison JB Le Negrate G Welsh K Huang Z Knoefel WT Reed JC XIAP mediates NOD signaling via interaction with RIP2 Proc Natl Acad Sci USA 2009 15 14524 14529 10.1073/pnas.0907131106 19667203 PMC2732880

[B48] Montfort A de Badts B Douin-Echinard V Martin PG Iacovoni J Nevoit C Therville N Garcia V Bertrand MA Bessières MH Trombe MC Levade T Benoist H Ségui B FAN stimulates TNF(alpha)-induced gene expression, leukocyte recruitment, and humoral response J Immunol 2009 15 5369 5378 10.4049/jimmunol.0803384 19786552

[B49] Cheung C Akiyama TE Kudo G Gonzalez FJ Hepatic expression of cytochrome P450s in hepatocyte nuclear factor 1-alpha (HNF1alpha)-deficient mice Biochem Pharmacol 2003 15 2011 2020 10.1016/S0006-2952(03)00586-0 14599559

[B50] Wang Z Bishop EP Burke PA Expression profile analysis of the inflammatory response regulated by hepatocyte nuclear factor 4 α BMC Genomics 2011 15 128 10.1186/1471-2164-12-128 21352552 PMC3053261

[B51] Li R Pei H Watson DK Regulation of Ets function by protein-protein interactions Oncogene 2000 15 6514 6523 10.1038/sj.onc.1204035 11175367

