# Supplementary material for: Genomic characterization of remission in juvenile idiopathic arthritis
Source: Arthritis Res Ther. 2013 Aug 30;15(4):R100. doi: 10.1186/ar4280 (PMC4062846; doi:10.1186/ar4280)
Supplement: Additional file 1 — Table S1. Differentially expressed genes in PBMC in JIA patients who achieved remission with methotrexate alone vs. controls. Genes listed more than once indicate different probes for the same gene which showed different values in expression. [file ar4280-S1.PDF]

A

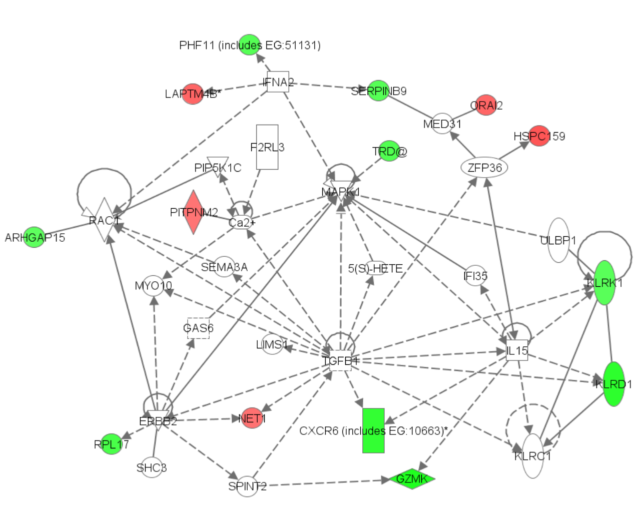
PHF11 (includes EG:51131)
IFNA2
LAPTM4B*
SERPINB9
ORAI2
MED31
HSPC159
F2RL3
TRD@
ZFP36
PIP5K1C
PITPNM2
Ca2+
MAPK
RAC1
ARHGAP15
ULBP1
KLRK1
SEMA3A
5(S)-HETE
IFI35
MYO10
GAS6
LIMS1
TGFB1
IL15
KLRD1
ERBB2
NET1
CXCR6 (includes EG:10663)*
RPL17
SHC3
SPINT2
GZMK
KLRC1

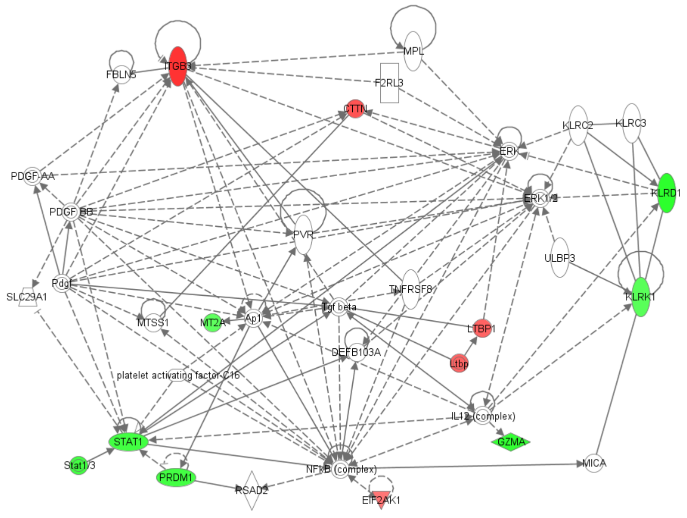
ITGB3
MPL
FBLN5
F2RL3
CTTN
KLRC2
KLRC3
ERK
PDGF-AA
PDGF BB
ERK1/2
KLRD1
PVR
ULBP3
Pdgf
SLC29A1
TNFRSF8
KLRK1
MTSS1
MT2A
Ap1
Tgf beta
LTBP1
DEFB103A
Ltbp
platelet activating factor-C16
IL12 (complex)
STAT1
GZMA
Stat1/3
NFkB (complex)
MICA
PRDM1
PSAD2
EIF2AK1

B

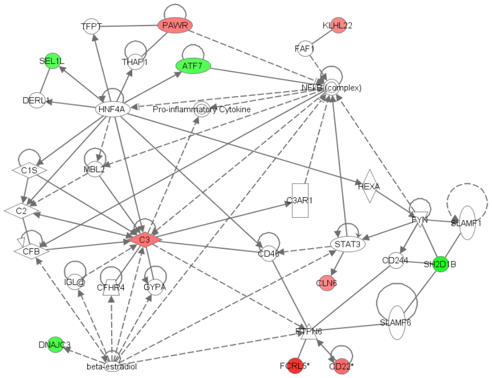
TFPT
PAWR
KLHL22
FAF1
SEL1L
THAP1
ATF7
NFkB (complex)
DERL1
HNF4A
Pro-inflammatory Cytokine
C1S
MBL2
HEXA
C2
C3AR1
FYN
SLAMF1
CFB
C3
CD46
STAT3
CD244
SH2D1B
IGL@
CFHR4
GYPA
CLN6
SLAMF6
DNAJC3
PTPN6
beta-estradiol
FCRL5*
CD22*

Supplemental Figure 1. Interactions between products of differentially expressed genes in PBMC from patients with JIA who achieved remission using methotrexate alone (A) or Etanercept and Methotrexate (B) relative to PBMC from controls. Differentially expressed genes entered in the Ingenuity Pathway Analysis program are colored. Genes shown in red show higher expression in patients compared with controls, and those shown in green show lower expression. Genes not colored were added by the IPA program to generate these networks.
