# Supplementary figures and images for: Genomic characterization of remission in juvenile idiopathic arthritis
Source: Arthritis Res Ther. 2013 Aug 30;15(4):R100. doi: 10.1186/ar4280 (PMC4062846; doi:10.1186/ar4280)

A

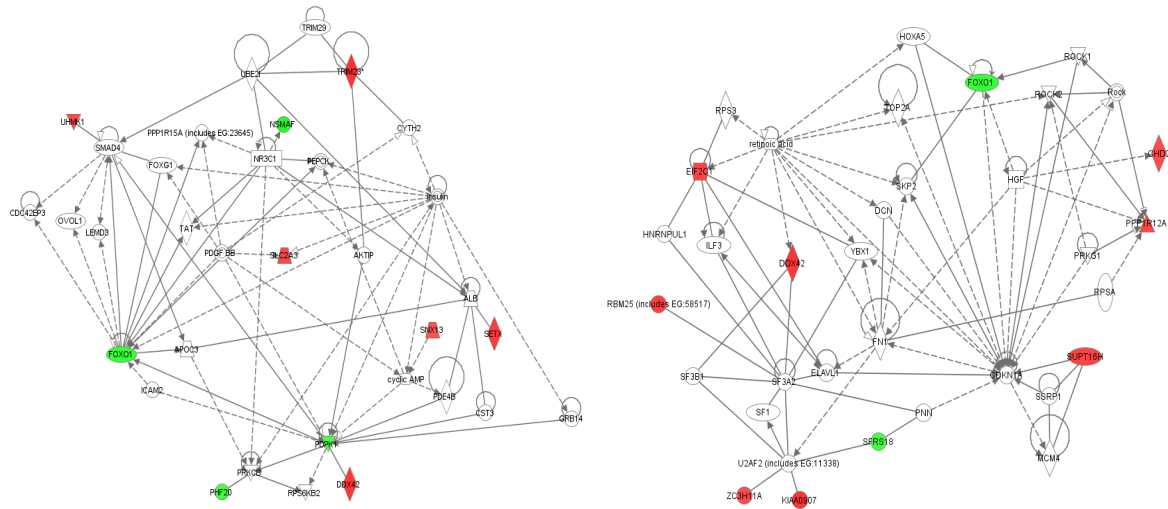

B

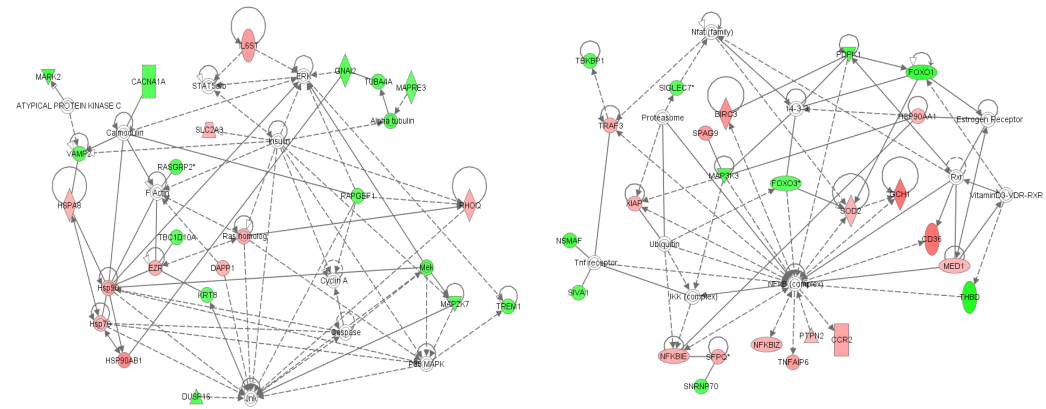

Supplement: Additional file 2 — Table S2. Differentially expressed genes in PBMC in JIA patients who achieved remission with etanercept and methotrexate vs. controls. Genes listed more than once indicate different probes for the same gene which showed different values in expression. [file ar4280-S2.PDF]
