# Supplementary material for: Genomic characterization of remission in juvenile idiopathic arthritis
Source: Arthritis Res Ther. 2013 Aug 30;15(4):R100. doi: 10.1186/ar4280 (PMC4062846; doi:10.1186/ar4280)
Supplement: Additional file 3 — Table S3. Differentially expressed genes in granulocytes of JIA patients who achieved remission with methotrexate and etanercept vs. controls. Genes listed more than once indicate different probes for the same gene which showed different values in expression. [file ar4280-S3.DOCX]

Supplemental Table 3. Differentially Expressed Genes in Granulocytes of JIA Patients Who Achieved Remission with Methotrexate and Etanercept vs Controls.

| **Gene Symbol** | **Gene Title** | **MTX+Et** | **Control** | **Fold-change**  **MTX+Et vs Control** | **Probe set** |
| --- | --- | --- | --- | --- | --- |
| ABCF1 | ATP-binding cassette, sub-family F (GCN20), member 1 | 161.98 | 120.01 | 1.35 | 200045_at |
| AFF1 | af4/fmr2 family, member 1 | 173.29 | 104.76 | 1.65 | 239661_at |
| ARAP2 | ArfGAP with RhoGAP domain, ankyrin repeat and PH domain 2 | 243.42 | 143.32 | 1.70 | 213618_at |
| ARF1 | ADP-ribosylation factor 1 | 189.59 | 287.79 | -1.52 | 1565651_at |
| ARL11 | ADP-ribosylation factor-like 11 | 641.94 | 418.55 | 1.53 | 1552691_at |
| ARMC8 | armadillo repeat containing 8 | 118.55 | 76.38 | 1.55 | 236966_at |
| ARSA | arylsulfatase A | 111.86 | 167.48 | -1.50 | 204443_at |
| ATM | ataxia telangiectasia mutated | 231.86 | 118.64 | 1.95 | 208442_s_at |
| ATM | ataxia telangiectasia mutated | 415.24 | 260.38 | 1.59 | 210858_x_at |
| BBX | bobby sox homolog (Drosophila) | 606.00 | 386.87 | 1.57 | 213016_at |
| BCKDK | branched chain ketoacid dehydrogenase kinase | 188.91 | 287.17 | -1.52 | 202030_at |
| BIRC3 | baculoviral IAP repeat-containing 3 | 254.39 | 120.46 | 2.11 | 210538_s_at |
| BIRC7 | baculoviral IAP repeat-containing 7 | 51.60 | 71.32 | -1.38 | 220451_s_at |
| BRD4 | bromodomain containing 4 | 928.07 | 1295.40 | -1.40 | 202102_s_at |
| BRD7 | bromodomain containing 7 | 171.07 | 111.06 | 1.54 | 222737_s_at |
| C14orf45 | chromosome 14 open reading frame 45 | 65.70 | 91.55 | -1.39 | 239933_x_at |
| C16orf48 | chromosome 16 open reading frame 48 | 58.79 | 92.28 | -1.57 | 223407_at |
| C16orf93 | chromosome 16 open reading frame 93 | 47.65 | 71.65 | -1.50 | 231300_at |
| C19orf6 | chromosome 19 open reading frame 6 | 63.10 | 100.72 | -1.60 | 212575_at |
| C1galt1 | core 1 synthase, glycoprotein-n-acetylgalactosamine 3-beta-galactosyltransferase, 1 | 395.55 | 229.19 | 1.73 | 226107_at |
| CACNA1A | calcium channel, voltage-dependent, P/Q type, alpha 1A subunit | 45.82 | 69.49 | -1.52 | 206399_x_at |
| CCBL2 | kynurenine aminotransferase iii | 197.71 | 119.15 | 1.66 | 237218_at |
| CCR2 | chemokine (C-C motif) receptor 2 | 72.46 | 41.26 | 1.76 | 207794_at |
| CD163 | CD163 molecule | 77.86 | 38.32 | 2.03 | 215049_x_at |
| CD36 | CD36 molecule (thrombospondin receptor) | 65.84 | 24.14 | 2.73 | 228766_at |
| CDC42SE2 | cdc42 small effector 2 | 224.91 | 126.39 | 1.78 | 234260_at |
| CEBPZ | ccaat/enhancer binding protein zeta | 86.67 | 59.04 | 1.47 | 229746_x_at |
| CHD2 | chromodomain helicase DNA binding protein 2 | 226.72 | 162.39 | 1.40 | 1554015_a_at |
| CIB1 | calcium and integrin binding 1 (calmyrin) | 449.11 | 666.89 | -1.48 | 201953_at |
| CMIP | c-Maf-inducing protein | 553.64 | 806.82 | -1.46 | 224992_s_at |
| CNPY2 | canopy 2 homolog (zebrafish) | 69.45 | 40.07 | 1.73 | 209797_at |
| CYBB | cytochrome b-245, beta polypeptide | 403.96 | 184.71 | 2.19 | 203922_s_at |
| CYBB | cytochrome b-245, beta polypeptide | 1189.48 | 589.63 | 2.02 | 203923_s_at |
| DAPK3 | death-associated protein kinase 3 | 83.52 | 122.84 | -1.47 | 203891_s_at |
| DAPP1 | dual adaptor of phosphotyrosine and 3-phosphoinositides | 2228.32 | 1511.51 | 1.47 | 222858_s_at |
| DCBLD2 | discoidin, CUB and LCCL domain containing 2 | 43.20 | 67.79 | -1.57 | 239446_x_at |
| DDX18 | DEAD (Asp-Glu-Ala-Asp) box polypeptide 18 | 78.05 | 49.22 | 1.59 | 205763_s_at |
| DLD | dihydrolipoamide dehydrogenase | 293.72 | 171.86 | 1.71 | 230426_at |
| DNAJC16 | DnaJ (Hsp40) homolog, subfamily C, member 16 | 92.77 | 60.15 | 1.54 | 212908_at |
| DOCK5 | dedicator of cytokinesis 5 | 98.71 | 35.74 | 2.76 | 1561276_at |
| DPM3 | dolichyl-phosphate mannosyltransferase polypeptide 3 | 81.86 | 113.64 | -1.39 | 219373_at |
| DUSP16 | dual specificity phosphatase 16 | 53.55 | 80.97 | -1.51 | 224336_s_at |
| EEF1A1 | eukaryotic translation elongation factor 1 alpha 1 | 170.61 | 94.76 | 1.80 | 227708_at |
| EFR3A | EFR3 homolog A (S. cerevisiae) | 244.96 | 152.76 | 1.60 | 212149_at |
| EGLN1 | egl nine homolog 1 (c. elegans) | 518.87 | 215.88 | 2.40 | 1561893_at |
| EIF4G3 | eukaryotic translation initiation factor 4 gamma, 3 | 236.43 | 149.31 | 1.58 | 201935_s_at |
| EML4 | echinoderm microtubule associated protein like 4 | 218.32 | 142.87 | 1.53 | 220386_s_at |
| EZR | ezrin | 139.72 | 88.47 | 1.58 | 217234_s_at |
| [FAM176B, LOC100133999] | family with sequence similarity 176, member B, hypothetical protein LOC100133999 | 111.78 | 161.54 | -1.45 | 221710_x_at |
| FCRL5 | Fc receptor-like 5 | 116.90 | 64.68 | 1.81 | 224406_s_at |
| FOXO1 | forkhead box O1 | 113.42 | 204.69 | -1.80 | 202723_s_at |
| [FOXO3, FOXO3B] | forkhead box O3, forkhead box O3B pseudogene | 507.31 | 730.54 | -1.44 | 210655_s_at |
| [FOXO3, FOXO3B] | forkhead box O3, forkhead box O3B pseudogene | 84.84 | 121.97 | -1.44 | 217399_s_at |
| [FUT5, NDUFA11] | fucosyltransferase 5 (alpha (1,3) fucosyltransferase), NADH dehydrogenase (ubiquinone) 1 alpha subcomplex, 11, 14.7kDa | 230.26 | 318.40 | -1.38 | 225304_s_at |
| FUZ | fuzzy homolog (Drosophila) | 87.45 | 147.41 | -1.69 | 221187_s_at |
| GBP1 | guanylate binding protein 1, interferon-inducible, 67kDa | 148.50 | 57.87 | 2.57 | 231578_at |
| GCH1 | GTP cyclohydrolase 1 | 758.46 | 279.32 | 2.72 | 204224_s_at |
| GEMIN7 | gem (nuclear organelle) associated protein 7 | 132.28 | 191.42 | -1.45 | 1555751_a_at |
| GEMIN7 | gem (nuclear organelle) associated protein 7 | 138.92 | 202.76 | -1.46 | 222821_s_at |
| GLS | glutaminase | 69.96 | 44.81 | 1.56 | 223079_s_at |
| GNAI2 | guanine nucleotide binding protein (G protein), alpha inhibiting activity polypeptide 2 | 1566.34 | 2422.75 | -1.55 | 201040_at |
| GNG2 | guanine nucleotide binding protein (g protein), gamma 2 | 203.54 | 387.52 | -1.90 | 243819_at |
| GNL3L | guanine nucleotide binding protein-like 3 (nucleolar)-like | 258.45 | 159.25 | 1.62 | 205010_at |
| GPX3 | glutathione peroxidase 3 (plasma) | 43.12 | 80.64 | -1.87 | 214091_s_at |
| HDHD1A | haloacid dehalogenase-like hydrolase domain containing 1A | 85.92 | 55.79 | 1.54 | 203974_at |
| HERC4 | hect domain and RLD 4 | 100.82 | 70.63 | 1.43 | 208055_s_at |
| HIST1H1C | histone cluster 1, H1c | 355.87 | 587.86 | -1.65 | 209398_at |
| HM13 | histocompatibility (minor) 13 | 374.29 | 550.08 | -1.47 | 224615_x_at |
| HNRNPU | heterogeneous nuclear ribonucleoprotein U (scaffold attachment factor A) | 82.01 | 59.42 | 1.38 | 225805_at |
| HSP90AA1 | heat shock protein 90kDa alpha (cytosolic), class A member 1 | 789.83 | 538.67 | 1.47 | 211968_s_at |
| HSP90AB1 | heat shock protein 90kDa alpha (cytosolic), class B member 1 | 210.22 | 92.98 | 2.26 | 1557910_at |
| HSPA8 | heat shock 70kDa protein 8 | 2231.00 | 1503.23 | 1.48 | 210338_s_at |
| IDS | iduronate 2-sulfatase | 1108.16 | 1573.44 | -1.42 | 206342_x_at |
| IER5 | immediate early response 5 | 1190.02 | 788.79 | 1.51 | 218611_at |
| IL6ST | interleukin 6 signal transducer (gp130, oncostatin M receptor) | 256.85 | 134.26 | 1.91 | 212196_at |
| KCNK7 | potassium channel, subfamily K, member 7 | 47.87 | 70.31 | -1.47 | 220412_x_at |
| KCNK7 | potassium channel, subfamily K, member 7 | 50.35 | 70.41 | -1.40 | 224008_s_at |
| KIAA0368 | KIAA0368 | 119.77 | 75.26 | 1.59 | 212428_at |
| KIAA0907 | KIAA0907 | 73.67 | 50.24 | 1.47 | 230028_at |
| KIAA0913 | KIAA0913 | 660.34 | 939.17 | -1.42 | 212359_s_at |
| KIAA1632 | KIAA1632 | 131.80 | 79.77 | 1.65 | 1563471_at |
| KIF27 | kinesin family member 27 | 86.65 | 144.84 | -1.67 | 240547_at |
| KRIT1 | KRIT1, ankyrin repeat containing | 135.26 | 52.25 | 2.59 | 34031_i_at |
| [KRT8, KRT8P9, LOC149501, LOC647954] | keratin 8, keratin 8 pseudogene 9, similar to keratin 8, similar to keratin 8 | 46.28 | 64.02 | -1.38 | 216821_at |
| LOC100129105 | similar to hCG1821214 | 67.24 | 96.61 | -1.44 | 231402_at |
| LOC254128 | hypothetical protein LOC254128 | 67.08 | 49.90 | 1.34 | 235132_at |
| LOC284454 | hypothetical protein LOC284454 | 313.08 | 487.61 | -1.56 | 1555847_a_at |
| LOC285949 | hypothetical protein LOC285949 | 176.67 | 97.89 | 1.80 | 1563781_at |
| LONP2 | lon peptidase 2, peroxisomal | 81.52 | 53.53 | 1.52 | 223098_s_at |
| Mad1l1 | mad1 mitotic arrest deficient-like 1 (yeast) | 83.78 | 33.39 | 2.51 | 233921_s_at |
| MAP2K7 | mitogen-activated protein kinase kinase 7 | 84.77 | 129.05 | -1.52 | 226023_at |
| MAP3K3 | mitogen-activated protein kinase kinase kinase 3 | 495.08 | 799.72 | -1.62 | 203514_at |
| MAP4K4 | mitogen-activated protein kinase kinase kinase kinase 4 | 410.35 | 237.63 | 1.73 | 238769_at |
| MAPK1 | mitogen-activated protein kinase 1 | 77.91 | 23.84 | 3.27 | 1562283_at |
| MAPRE3 | microtubule-associated protein, RP/EB family, member 3 | 75.26 | 104.21 | -1.38 | 203842_s_at |
| MARK2 | MAP/microtubule affinity-regulating kinase 2 | 272.04 | 449.02 | -1.65 | 203942_s_at |
| MCOLN1 | mucolipin 1 | 140.54 | 243.77 | -1.73 | 219952_s_at |
| MDM4 | Mdm4 p53 binding protein homolog (mouse) | 162.40 | 73.88 | 2.20 | 225742_at |
| MED1 | mediator complex subunit 1 | 166.89 | 117.48 | 1.42 | 225452_at |
| MGAT4A | mannosyl (alpha-1,3-)-glycoprotein beta-1,4-N-acetylglucosaminyltransferase, isozyme A | 118.65 | 81.84 | 1.45 | 231283_at |
| MGC2752 | hypothetical LOC65996 | 235.95 | 379.36 | -1.61 | 218624_s_at |
| MITD1 | MIT, microtubule interacting and transport, domain containing 1 | 233.98 | 152.71 | 1.53 | 226329_s_at |
| MRPL45 | mitochondrial ribosomal protein L45 | 77.39 | 52.71 | 1.47 | 224479_s_at |
| MYADM | myeloid-associated differentiation marker | 503.35 | 796.32 | -1.58 | 224920_x_at |
| MYH7B | myosin, heavy polypeptide 7b, cardiac muscle, beta | 96.91 | 60.77 | 1.59 | 1557744_at |
| MYST1 | MYST histone acetyltransferase 1 | 95.21 | 148.38 | -1.56 | 214885_at |
| NBN | nibrin | 1160.69 | 650.26 | 1.78 | 202905_x_at |
| NBN | nibrin | 1523.54 | 858.96 | 1.77 | 202906_s_at |
| NBN | nibrin | 1181.14 | 648.79 | 1.82 | 202907_s_at |
| NBN | nibrin | 471.09 | 256.48 | 1.84 | 217299_s_at |
| NBN | Nibrin | 118.02 | 66.09 | 1.79 | 240510_at |
| NCAPH2 | non-SMC condensin II complex, subunit H2 | 48.44 | 69.97 | -1.44 | 205086_s_at |
| NCRNA00084 | non-protein coding RNA 84 | 2438.77 | 3590.42 | -1.47 | 224566_at |
| NFKBIE | nuclear factor of kappa light polypeptide gene enhancer in B-cells inhibitor, epsilon | 155.57 | 100.50 | 1.55 | 203927_at |
| NFKBIZ | nuclear factor of kappa light polypeptide gene enhancer in B-cells inhibitor, zeta | 2928.41 | 1955.44 | 1.50 | 223217_s_at |
| NSMAF | Neutral sphingomyelinase (N-SMase) activation associated factor | 94.05 | 153.99 | -1.64 | 232148_at |
| NUFIP2 | nuclear fragile x mental retardation protein interacting protein 2 | 80.48 | 138.81 | -1.72 | 243394_at |
| OAZ2 | ornithine decarboxylase antizyme 2 | 1883.10 | 2735.61 | -1.45 | 201364_s_at |
| OAZ2 | ornithine decarboxylase antizyme 2 | 1188.32 | 1641.74 | -1.38 | 201365_at |
| ODF3B | outer dense fiber of sperm tails 3B | 54.89 | 72.10 | -1.31 | 238325_s_at |
| PATL1 | protein associated with topoisomerase II homolog 1 (yeast) | 107.48 | 74.18 | 1.45 | 244342_at |
| PCIF1 | PDX1 C-terminal inhibiting factor 1 | 99.36 | 148.30 | -1.49 | 222045_s_at |
| PDLIM2 | PDZ and LIM domain 2 (mystique) | 422.08 | 693.29 | -1.64 | 219165_at |
| PDPK1 | 3-phosphoinositide dependent protein kinase-1 | 382.91 | 606.99 | -1.59 | 204524_at |
| PDS5A | scc-112 protein | 77.80 | 52.72 | 1.48 | 241798_at |
| PGP | phosphoglycolate phosphatase | 104.21 | 158.56 | -1.52 | 222622_at |
| PHF17 | PHD finger protein 17 | 165.56 | 268.77 | -1.62 | 225820_at |
| PHF20 | PHD finger protein 20 | 440.00 | 617.95 | -1.40 | 206567_s_at |
| PLXNC1 | plexin C1 | 1764.40 | 1229.47 | 1.44 | 206470_at |
| PNPLA2 | patatin-like phospholipase domain containing 2 | 142.73 | 194.58 | -1.36 | 212705_x_at |
| PPA2 | pyrophosphatase (inorganic) 2 | 255.77 | 183.83 | 1.39 | 220741_s_at |
| Prdm2 | pr domain containing 2, with znf domain | 65.96 | 44.14 | 1.49 | 239655_at |
| PRG2 | plasticity-related gene 2 | 45.71 | 65.27 | -1.43 | 220798_x_at |
| PTPN2 | protein tyrosine phosphatase, non-receptor type 2 | 76.43 | 47.02 | 1.63 | 1557193_at |
| PTPN2 | protein tyrosine phosphatase, non-receptor type 2 | 178.97 | 131.78 | 1.36 | 213137_s_at |
| RAB11FIP4 | RAB11 family interacting protein 4 (class II) | 155.02 | 95.57 | 1.62 | 225746_at |
| RAB5C | RAB5C, member RAS oncogene family | 500.35 | 816.89 | -1.63 | 201140_s_at |
| RAB5C | RAB5C, member RAS oncogene family | 474.54 | 768.06 | -1.62 | 201156_s_at |
| RAB7L1 | RAB7, member RAS oncogene family-like 1 | 210.57 | 123.10 | 1.71 | 218700_s_at |
| RALGPS2 | Ral GEF with PH domain and SH3 binding motif 2 | 64.24 | 36.72 | 1.75 | 220338_at |
| RAP2B | RAP2B, member of RAS oncogene family | 274.52 | 192.37 | 1.43 | 213923_at |
| RAPGEF1 | Rap guanine nucleotide exchange factor (GEF) 1 | 64.48 | 93.90 | -1.46 | 204543_at |
| RASGRP2 | RAS guanyl releasing protein 2 (calcium and DAG-regulated) | 65.18 | 95.88 | -1.47 | 214368_at |
| RASGRP2 | RAS guanyl releasing protein 2 (calcium and DAG-regulated) | 338.32 | 511.39 | -1.51 | 214369_s_at |
| RBM25 | RNA binding motif protein 25 | 439.59 | 299.25 | 1.47 | 1557081_at |
| RFX5 | regulatory factor X, 5 (influences HLA class II expression) | 109.93 | 76.48 | 1.44 | 202964_s_at |
| RHOQ | ras homolog gene family, member Q | 831.29 | 530.57 | 1.57 | 1559582_at |
| RNF125 | ring finger protein 125 | 69.66 | 36.61 | 1.90 | 207735_at |
| rnf145 | hypothetical protein flj31951 | 576.95 | 332.27 | 1.74 | 236322_at |
| RNF145 | ring finger protein 145 | 326.26 | 520.03 | -1.59 | 238949_at |
| RNF167 | ring finger protein 167 | 645.80 | 949.55 | -1.47 | 212047_s_at |
| RNF24 | ring finger protein 24 | 215.52 | 85.97 | 2.51 | 1562307_at |
| RP11-298P3.3 | phosphonoformate immuno-associated protein 5 | 187.64 | 66.81 | 2.81 | 1565743_at |
| RSBN1 | round spermatid basic protein 1 | 103.88 | 154.27 | -1.49 | 222788_s_at |
| RUFY2 | RUN and FYVE domain containing 2 | 787.71 | 1203.91 | -1.53 | 219957_at |
| SAPS3 | SAPS domain family member 3 | 254.80 | 389.05 | -1.53 | 228105_at |
| SEC14L1 | SEC14-like 1 (S. cerevisiae) | 1957.88 | 2773.35 | -1.42 | 202083_s_at |
| SFPQ | splicing factor proline/glutamine-rich (polypyrimidine tract binding protein associated) | 243.48 | 166.63 | 1.46 | 201585_s_at |
| SFPQ | splicing factor proline/glutamine-rich (polypyrimidine tract binding protein associated) | 574.51 | 344.65 | 1.67 | 201586_s_at |
| SFRS18 | splicing factor, arginine/serine-rich 18 | 322.09 | 439.89 | -1.37 | 226412_at |
| SFRS3 | splicing factor, arginine/serine-rich 3 | 127.66 | 78.40 | 1.63 | 235324_at |
| SIGLEC7 | sialic acid binding Ig-like lectin 7 | 171.01 | 252.07 | -1.47 | 216537_s_at |
| SIGLEC7 | sialic acid binding Ig-like lectin 7 | 211.57 | 302.60 | -1.43 | 217159_x_at |
| SIVA1 | SIVA1, apoptosis-inducing factor | 108.89 | 160.33 | -1.47 | 210792_x_at |
| SLAMF7 | SLAM family member 7 | 85.95 | 41.94 | 2.05 | 222838_at |
| SLC2A3 | solute carrier family 2 (facilitated glucose transporter), member 3 | 1308.52 | 875.21 | 1.50 | 202498_s_at |
| SLC35B2 | solute carrier family 35, member B2 | 112.20 | 74.25 | 1.51 | 224716_at |
| SLFN5 | schlafen family member 5 | 87.00 | 34.11 | 2.55 | 243999_at |
| SNRNP70 | small nuclear ribonucleoprotein 70kDa (U1) | 45.19 | 69.62 | -1.54 | 213121_at |
| SOD2 | superoxide dismutase 2, mitochondrial | 5909.47 | 4067.02 | 1.45 | 221477_s_at |
| SPAG9 | sperm associated antigen 9 | 413.77 | 214.38 | 1.93 | 1554543_at |
| [SULT1A3, SULT1A4] | sulfotransferase family, cytosolic, 1A, phenol-preferring, member 3, sulfotransferase family, cytosolic, 1A, phenol-preferring, member 4 | 390.99 | 539.46 | -1.38 | 209607_x_at |
| TAGLN2 | transgelin 2 | 4930.51 | 7417.34 | -1.50 | 200916_at |
| TAGLN2 | transgelin 2 | 2805.26 | 4831.62 | -1.72 | 210978_s_at |
| TBC1D10A | TBC1 domain family, member 10A | 194.09 | 278.24 | -1.43 | 226133_s_at |
| TBKBP1 | TBK1 binding protein 1 | 78.77 | 117.80 | -1.50 | 205424_at |
| TBXAS1 | thromboxane A synthase 1 (platelet) | 521.10 | 829.20 | -1.59 | 208130_s_at |
| TCTN1 | tectonic family member 1 | 100.38 | 144.74 | -1.44 | 218584_at |
| TEP1 | telomerase-associated protein 1 | 74.35 | 41.63 | 1.79 | 205727_at |
| TGFBR1 | transforming growth factor beta receptor I | 116.43 | 83.35 | 1.40 | 239605_x_at |
| THBD | thrombomodulin | 185.84 | 377.61 | -2.03 | 203888_at |
| TMEM140 | transmembrane protein 140 | 579.53 | 941.67 | -1.62 | 243465_at |
| [TMEM192, ZNF320] | transmembrane protein 192, zinc finger protein 320 | 67.55 | 42.78 | 1.58 | 1555790_a_at |
| TMEM63A | transmembrane protein 63A | 142.54 | 92.84 | 1.54 | 214833_at |
| TMEM77 | transmembrane protein 77 | 68.92 | 43.28 | 1.59 | 225228_at |
| TMEM87B | transmembrane protein 87B | 103.42 | 63.38 | 1.63 | 225412_at |
| TNFAIP6 | tumor necrosis factor, alpha-induced protein 6 | 2201.76 | 1159.48 | 1.90 | 206026_s_at |
| TRAF3 | TNF receptor-associated factor 3 | 113.85 | 72.26 | 1.58 | 221571_at |
| TREM1 | triggering receptor expressed on myeloid cells 1 | 2700.19 | 4000.42 | -1.48 | 219434_at |
| TRIM23 | tripartite motif-containing 23 | 84.42 | 55.05 | 1.53 | 210995_s_at |
| TRIM4 | tripartite motif-containing 4 | 137.52 | 79.16 | 1.74 | 223384_s_at |
| TRMT2B | TRM2 tRNA methyltransferase 2 homolog B (S. cerevisiae) | 64.97 | 42.69 | 1.52 | 205238_at |
| TST | thiosulfate sulfurtransferase (rhodanese) | 436.72 | 606.18 | -1.39 | 209605_at |
| TUBA4A | tubulin, alpha 4a | 1552.30 | 2349.43 | -1.51 | 212242_at |
| UBA6 | ubiquitin-like modifier activating enzyme 6 | 177.26 | 131.05 | 1.35 | 222600_s_at |
| UBA6 | ubiquitin-like modifier activating enzyme 6 | 103.91 | 75.06 | 1.38 | 222601_at |
| ULK1 | unc-51-like kinase 1 (c. elegans) | 546.95 | 792.84 | -1.45 | 238042_at |
| USP47 | ubiquitin specific peptidase 47 | 93.45 | 54.90 | 1.70 | 223701_s_at |
| VAMP2 | vesicle-associated membrane protein 2 (synaptobrevin 2) | 199.67 | 289.14 | -1.45 | 201557_at |
| VDAC1 | voltage-dependent anion channel 1 | 89.87 | 48.37 | 1.86 | 212038_s_at |
| WTAP | wilms tumor 1 associated protein | 218.28 | 143.69 | 1.52 | 244219_at |
| XIAP | X-linked inhibitor of apoptosis | 293.21 | 189.35 | 1.55 | 243026_x_at |
| XIST | X (inactive)-specific transcript (non-protein coding) | 94.04 | 23.58 | 3.99 | 224589_at |
| XRN1 | 5'-3' exoribonuclease 1 | 137.74 | 78.63 | 1.75 | 1555785_a_at |
| ZC3H7A | zinc finger CCCH-type containing 7A | 241.81 | 173.21 | 1.40 | 226897_s_at |
| ZNF277 | zinc finger protein 277 | 112.76 | 73.93 | 1.53 | 218645_at |
| ZNF281 | zinc finger protein 281 | 537.49 | 764.74 | -1.42 | 218401_s_at |
| ZNF324 | zinc finger protein 324 | 117.04 | 181.73 | -1.55 | 205182_s_at |
| ZNF330 | zinc finger protein 330 | 163.70 | 117.79 | 1.39 | 209814_at |
| ZNF646 | zinc finger protein 646 | 62.00 | 85.72 | -1.38 | 204876_at |
| --- | --- | 292.00 | 154.20 | 1.89 | 1557270_at |
| --- | --- | 225.71 | 124.49 | 1.81 | 1558515_at |
| --- | --- | 335.28 | 474.28 | -1.41 | 1559467_at |
| --- | --- | 80.94 | 42.65 | 1.90 | 1569538_at |
| --- | --- | 268.10 | 193.33 | 1.39 | 214808_at |
| --- | --- | 205.59 | 100.31 | 2.05 | 215029_at |
| --- | --- | 124.02 | 78.45 | 1.58 | 224082_at |
| --- | --- | 478.76 | 716.83 | -1.50 | 228528_at |
| --- | --- | 236.35 | 177.97 | 1.33 | 229319_at |
| --- | --- | 828.00 | 423.78 | 1.95 | 234987_at |
| --- | --- | 579.93 | 362.59 | 1.60 | 235028_at |
| --- | --- | 53.57 | 73.42 | -1.37 | 238141_s_at |
| --- | --- | 154.39 | 102.27 | 1.51 | 238918_at |
| --- | --- | 338.53 | 195.59 | 1.73 | 239464_at |
| --- | --- | 109.54 | 162.21 | -1.48 | 242139_s_at |
