# Supplementary material for: Genomic characterization of remission in juvenile idiopathic arthritis
Source: Arthritis Res Ther. 2013 Aug 30;15(4):R100. doi: 10.1186/ar4280 (PMC4062846; doi:10.1186/ar4280)
Supplement: Additional file 4 — Table S4. Differentially expressed genes in granulocytes of JIA patients who achieved remission with methotrexate alone vs. controls. Genes listed more than once indicate different probes for the same gene which showed different values in expression. [file ar4280-S4.DOCX]

Supplemental Table 4. Differentially Expressed Genes in Granulocytes of JIA Patients Who Achieved Remission with Methotrexate alone vs Controls.

| **Gene Symbol** | **Gene Title** | **Mtx** | **Control** | **Fold-change**  **Mtx v Control** | **Probe set** |
| --- | --- | --- | --- | --- | --- |
| CDV3 | CDV3 homolog (mouse) | 221.31 | 149.93 | 1.48 | 213548_s_at |
| CHD2 | chromodomain helicase DNA binding protein 2 | 222.48 | 162.39 | 1.37 | 1554015_a_at |
| DDX42 | DEAD (Asp-Glu-Ala-Asp) box polypeptide 42 | 97.61 | 63.62 | 1.53 | 1559954_s_at |
| EIF2C1 | eukaryotic translation initiation factor 2C, 1 | 131.10 | 90.83 | 1.44 | 222576_s_at |
| FOXO1 | forkhead box O1 | 131.00 | 204.69 | -1.56 | 202723_s_at |
| HEXIM1 | hexamethylene bis-acetamide inducible 1 | 113.57 | 161.52 | -1.42 | 214188_at |
| KIAA0907 | KIAA0907 | 77.93 | 50.24 | 1.55 | 230028_at |
| NSMAF | Neutral sphingomyelinase (N-SMase) activation associated factor | 89.13 | 153.99 | -1.73 | 232148_at |
| PDPK1 | 3-phosphoinositide dependent protein kinase-1 | 426.64 | 606.99 | -1.42 | 204524_at |
| PHF20 | PHD finger protein 20 | 425.51 | 617.95 | -1.45 | 206567_s_at |
| PPP1R12A | protein phosphatase 1, regulatory (inhibitor) subunit 12A | 1140.36 | 791.69 | 1.44 | 201602_s_at |
| RBM25 | RNA binding motif protein 25 | 443.83 | 299.25 | 1.48 | 1557081_at |
| saps3 | SAPS domain family member 3 | 217.92 | 389.05 | -1.79 | 228105_at |
| SETX | senataxin | 156.01 | 105.06 | 1.48 | 232229_at |
| SFRS18 | splicing factor, arginine/serine-rich 18 | 304.06 | 439.89 | -1.45 | 226412_at |
| [SLC2A14 /// SLC2A3] | solute carrier family 2 (facilitated glucose transporter), member 14 /// solute carrier family 2 (facilitated glucose transporter), member 3 | 1726.26 | 1223.51 | 1.41 | 216236_s_at |
| SNX13 | sorting nexin 13 | 73.07 | 55.17 | 1.32 | 215820_x_at |
| SUPT16H | suppressor of Ty 16 homolog (S. cerevisiae) | 77.70 | 53.64 | 1.45 | 233827_s_at |
| TMEM140 | transmembrane protein 140 | 546.44 | 941.67 | -1.72 | 243465_at |
| TRIM23 | tripartite motif-containing 23 | 128.41 | 85.26 | 1.51 | 210994_x_at |
| TRIM23 | tripartite motif-containing 23 | 85.87 | 55.05 | 1.56 | 210995_s_at |
| TTC17 | tetratricopeptide repeat domain 17 | 72.03 | 104.56 | -1.45 | 224852_at |
| UHMK1 | U2AF homology motif (UHM) kinase 1 | 172.94 | 121.96 | 1.42 | 235003_at |
| ZC3H11A | zinc finger CCCH-type containing 11A | 276.47 | 204.75 | 1.35 | 205787_x_at |
