# Supplementary material for: Genomic characterization of remission in juvenile idiopathic arthritis
Source: Arthritis Res Ther. 2013 Aug 30;15(4):R100. doi: 10.1186/ar4280 (PMC4062846; doi:10.1186/ar4280)
Supplement: Additional file 5 — Table S5. Differentially expressed genes in PBMC in JIA patients who achieved remission with methotrexate and etanercept vs. methotrexate alone. [file ar4280-S5.DOCX]

Supplemental Table 5. Differentially Expressed Genes in PBMC in JIA Patients Who Achieved Remission with Methotrexate and Etanercept vs Methotrexate alone

| Gene Symbol | Gene Title | MTX+Et | MTX | Fold-change MTX+Et v MTX | Probe set |
| --- | --- | --- | --- | --- | --- |
| CLCF1 | cardiotrophin-like cytokine factor 1 | 166.59 | 120.17 | 1.39 | 1556385_at |
| IGF1R | insulin-like growth factor 1 receptor | 87.11 | 119.08 | -1.37 | 203627_at |
| PRKY | protein kinase, Y-linked | 37.41 | 85.12 | -2.28 | 206279_at |
| C3 | complement component 3 | 140.50 | 75.74 | 1.86 | 217767_at |
| TSIX | XIST antisense RNA (non-protein coding) | 66.82 | 26.83 | 2.49 | 231592_at |
| --- | --- | 121.88 | 72.15 | 1.69 | 240861_at |
|  |  |  |  |  |  |
