# Supplementary material for: Genomic characterization of remission in juvenile idiopathic arthritis
Source: Arthritis Res Ther. 2013 Aug 30;15(4):R100. doi: 10.1186/ar4280 (PMC4062846; doi:10.1186/ar4280)
Supplement: Additional file 6 — Table S6. Differentially expressed genes in granulocytes in JIA patients who achieved remission with methotrexate and etanercept vs. methotrexate alone. Genes listed more than once indicate different probes for the same gene which showed different values in expression. [file ar4280-S6.DOCX]

Supplemental Table 6. Differentially Expressed Genes in Granulocytes in JIA Patients Who Achieved Remission with Methotrexate and Etanercept vs Methotrexate alone

| **Gene Symbol** | **Gene Title** | **MTX+Et** | **MTX** | **Fold-change MTX+Et vs MTX** | **Probe set** |
| --- | --- | --- | --- | --- | --- |
| ABTB1 | ankyrin repeat and BTB (POZ) domain containing 1 | 971.73 | 1401.44 | -1.44 | 226442_at |
| ANGPT1 | angiopoietin 1 | 47.05 | 99.97 | -2.12 | 205608_s_at |
| ARHGEF3 | Rho guanine nucleotide exchange factor (GEF) 3 | 298.54 | 181.19 | 1.65 | 218501_at |
| BIRC3 | baculoviral IAP repeat-containing 3 | 254.39 | 108.77 | 2.34 | 210538_s_at |
| BTNL8 | butyrophilin-like 8 | 646.91 | 119.43 | 5.42 | 220421_at |
| CCNG1 | cyclin G1 | 308.99 | 211.52 | 1.46 | 208796_s_at |
| CLIC4 | chloride intracellular channel 4 | 201.49 | 81.19 | 2.48 | 201560_at |
| CX3CR1 | chemokine (C-X3-C motif) receptor 1 | 1352.05 | 698.59 | 1.94 | 205898_at |
| CYBB | cytochrome b-245, beta polypeptide | 1189.48 | 584.64 | 2.03 | 203923_s_at |
| [DDIT3 /// NR1H3] | DNA-damage-inducible transcript 3 /// nuclear receptor subfamily 1, group H, member 3 | 558.79 | 784.79 | -1.40 | 209383_at |
| DDX3Y | DEAD (Asp-Glu-Ala-Asp) box polypeptide 3, Y-linked | 11.87 | 191.71 | -16.16 | 205000_at |
| DNAJC10 | DnaJ (Hsp40) homolog, subfamily C, member 10 | 134.68 | 73.39 | 1.84 | 225174_at |
| EIF1AY | eukaryotic translation initiation factor 1A, Y-linked | 10.16 | 214.64 | -21.13 | 204409_s_at |
| EIF1AY | eukaryotic translation initiation factor 1A, Y-linked | 10.74 | 113.11 | -10.53 | 204410_at |
| EIF1AY | eukaryotic translation initiation factor 1a, y-linked | 8.38 | 87.36 | -10.43 | 244482_at |
| ESD | esterase D/formylglutathione hydrolase | 278.93 | 182.35 | 1.53 | 209009_at |
| HDHD1A | haloacid dehalogenase-like hydrolase domain containing 1A | 85.92 | 54.22 | 1.58 | 203974_at |
| HIST1H1C | histone cluster 1, H1c | 355.87 | 625.01 | -1.76 | 209398_at |
| HIST1H2BF | histone cluster 1, H2bf | 412.67 | 613.53 | -1.49 | 208490_x_at |
| HLA-DOB | major histocompatibility complex, class II, DO beta | 133.20 | 72.67 | 1.83 | 205671_s_at |
| IGF1R | insulin-like growth factor 1 receptor | 1321.89 | 2055.06 | -1.55 | 203628_at |
| IL6ST | interleukin 6 signal transducer (gp130, oncostatin M receptor) | 599.25 | 308.41 | 1.94 | 212195_at |
| KIAA1683 | KIAA1683 | 70.01 | 93.96 | -1.34 | 223600_s_at |
| MYCL1 | v-myc myelocytomatosis viral oncogene homolog 1, lung carcinoma derived (avian) | 90.37 | 62.77 | 1.44 | 214058_at |
| NBN | nibrin | 1181.14 | 669.20 | 1.76 | 202907_s_at |
| NBN | nibrin | 471.09 | 264.46 | 1.78 | 217299_s_at |
| NBN | nibrin | 1523.54 | 852.71 | 1.79 | 202906_s_at |
| PION | pigeon homolog (Drosophila) | 239.86 | 119.57 | 2.01 | 222150_s_at |
| PRPF4B | PRP4 pre-mRNA processing factor 4 homolog B (yeast) | 190.28 | 134.30 | 1.42 | 202126_at |
| RBMX | RNA binding motif protein, X-linked | 335.64 | 240.80 | 1.39 | 213762_x_at |
| TMEM77 | transmembrane protein 77 | 216.70 | 144.93 | 1.50 | 225230_at |
| TNFAIP3 | tumor necrosis factor, alpha-induced protein 3 | 1164.22 | 712.15 | 1.63 | 202644_s_at |
| TRAF3 | TNF receptor-associated factor 3 | 113.85 | 69.29 | 1.64 | 221571_at |
| XIST | X (inactive)-specific transcript (non-protein coding) | 94.04 | 17.10 | 5.50 | 224589_at |
| XIST | X (inactive)-specific transcript (non-protein coding) | 175.39 | 27.28 | 6.43 | 221728_x_at |
| XIST | X (inactive)-specific transcript (non-protein coding) | 181.17 | 20.63 | 8.78 | 214218_s_at |
| XIST | X (inactive)-specific transcript (non-protein coding) | 229.31 | 19.80 | 11.58 | 224590_at |
| XIST | X (inactive)-specific transcript (non-protein coding) | 567.32 | 31.47 | 18.03 | 227671_at |
| XIST | X (inactive)-specific transcript (non-protein coding) | 1231.03 | 51.29 | 24.00 | 224588_at |
| ZNF644 | zinc finger protein 644 | 70.02 | 44.71 | 1.57 | 222580_at |
| --- | --- | 133.34 | 79.47 | 1.68 | 238604_at |
| --- | --- | 136.70 | 79.57 | 1.72 | 227682_at |
